# SALL1 functions as a tumor suppressor in breast cancer by regulating cancer cell senescence and metastasis through the NuRD complex

**DOI:** 10.1186/s12943-018-0824-y

**Published:** 2018-04-06

**Authors:** Chunling Ma, Fang Wang, Bing Han, Xiaoli Zhong, Fusheng Si, Jian Ye, Eddy C. Hsueh, Lynn Robbins, Susan M. Kiefer, Yanping Zhang, Pamela Hunborg, Mark A. Varvares, Michael Rauchman, Guangyong Peng

**Affiliations:** 1 Department of Internal Medicine, Saint Louis University School of Medicine, Saint Louis, MO 63104 USA; 2 Department of Laboratory Medicine, Women & Children’s Hospital of Linyi, Shandong Medical College, Linyi, 276000 People’s Republic of China; 3 Department of Laboratory Medicine, The First Affiliated Hospital of Nanjing Medical University, Nanjing, 210029 People’s Republic of China; 4 Department of Obstetrics and Gynecology, Qilu Hospital of Shandong University, Jinan, 250012 People’s Republic of China; 5 Department of Surgery, Saint Louis University School of Medicine, Saint Louis, MO 63104 USA; 6 VA Saint Louis Health Care System, John Cochran Division, St. Louis, MO 63106 USA; 7 Department of Medicine, Washington University, Saint. Louis, MO 63110 USA; 8 Department of Otolaryngology, Saint Louis University School of Medicine, Saint Louis, MO 63110 USA; 9 Department of Otolaryngology, Harvard Medical School, Boston, MA 02114 USA

**Keywords:** SALL1, Tumor suppressor gene, Senescence, NuRD complex, Tumorigenesis, Metastasis, Breast cancer

## Abstract

**Background:**

SALL1 is a multi-zinc finger transcription factor that regulates organogenesis and stem cell development, but the role of SALL1 in tumor biology and tumorigenesis remains largely unknown.

**Methods:**

We analyzed SALL1 expression levels in human and murine breast cancer cells as well as cancer tissues from different types of breast cancer patients. Using both in vitro co-culture system and in vivo breast tumor models, we investigated how SALL1 expression in breast cancer cells affects tumor cell growth and proliferation, metastasis, and cell fate. Using the gain-of function and loss-of-function strategies, we dissected the molecular mechanism responsible for SALL1 tumor suppressor functions.

**Results:**

We demonstrated that SALL1 functions as a tumor suppressor in breast cancer, which is significantly down-regulated in the basal like breast cancer and in estrogen receptor (ER), progesterone receptor (PR) and epidermal growth factor receptor 2 (HER2) triple negative breast cancer patients. SALL1 expression in human and murine breast cancer cells inhibited cancer cell growth and proliferation, metastasis, and promoted cell cycle arrest. Knockdown of SALL1 in breast cancer cells promoted cancer cell growth, proliferation, and colony formation. Our studies revealed that tumor suppression was mediated by recruitment of the Nucleosome Remodeling and Deacetylase (NuRD) complex by SALL1, which promoted cancer cell senescence. We further demonstrated that the mechanism of inhibition of breast cancer cell growth and invasion by SALL1-NuRD depends on the p38 MAPK, ERK1/2, and mTOR signaling pathways.

**Conclusion:**

Our studies indicate that the developmental control gene SALL1 plays a critical role in tumor suppression by recruiting the NuRD complex and thereby inducing cell senescence in breast cancer cells.

**Electronic supplementary material:**

The online version of this article (10.1186/s12943-018-0824-y) contains supplementary material, which is available to authorized users.

## Background

The human SALL gene family, SALL1-SALL4, was identified as homologues to the Drosophila homeotic gene *spalt* [1–3]. Originally, the SALL family are zinc finger transcription factors that were shown to function as critical regulators in the development of multiple mammalian organs, including kidney, heart, and the hematopoietic system [4–6]. Mutations in the human SALL1 and SALL4 genes result in Townes-Brocks (TBS) and Okihiro syndrome (OS), respectively [1, 5, 7]. The *SALL* gene family is also important for the control of stem cell pluripotency, differentiation and self-renewal properties involving transcriptional and epigenetic actions [6, 8–10].

Besides the regulation of organ and stem cell development, the role of SALL genes in tumor biology and tumorigenesis has been recently investigated. SALL2 has been reported as a potential tumor suppressor in ovarian cancer and Wilms tumor [11–13]. SALL4 was shown to regulate survival and apoptosis in human leukemic cells [14, 15]. Furthermore, SALL4 was recently identified as a novel marker for hepatoblastoma, non-small cell lung carcinoma, and gastric cancinoma [16, 17]. Mutations in SALL3 have been discovered in a significant proportion of Burkitt’s lymphoma cases [18]. It has been shown that the SALL1 promoter was methylated in breast and other epithelial cancers [19], but little is known about the role of SALL1 in the pathogenesis of human cancers. A recent report identified SALL1 as a tumor suppressor in human breast cancer, using an in vivo RNAi screen strategy [20]. However, the molecular mechanism and causative role of SALL1 in the regulation of breast cancer development and tumorigenesis are not well understood.

The role of SALL1 in the regulation of organogenesis of the kidney has been extensively studied by our group and others. We have demonstrated that SALL1 recruits and binds to the nucleosome remodeling and deacetylase (NuRD) chromatin remodeling complex and their combined action is required to maintain renal progenitor cells [4, 6, 21–23]. We identified a highly conserved 12-amino acid motif in the SALL1 that is sufficient for the recruitment of NuRD [22]. We showed that protein kinase C phosphorylates serine 2 of SALL1 repression motif to regulate SALL1-mediated NuRD recruitment and its associated functions [21]. Importantly, increasing evidence suggests that the NuRD protein complex plays an essential role in cancer development and metastasis [24]. Specifically, several subunits of NuRD, such as MTA1, MTA3, and Mi-2 can directly control the cancer invasive growth, epithelial-to-mesenchymal transition, and metastasis in breast cancer [24–26]. Given the recent study identifying that SALL1 could be a tumor suppressor in human breast cancer [20], it is important to determine how SALL1 regulates breast cancer cell biology and functions. In addition, whether SALL1 recruits the NuRD complex to perform its tumor suppressor function in breast cancer is unclear. Improved understanding of these molecular processes mediated by SALL1 for the regulation of tumor biology and tumorigenesis will open new avenues to develop novel therapeutic strategies in human breast cancer and possibly other tumors.

To better understand the role of SALL1 in the pathogenesis of breast cancer, we investigated the mechanism of SALL1 tumor suppressor activity in breast cancer models. Using both gain-of function and loss-of-function strategies, we showed that SALL1 expression in breast cancer cells inhibited tumor cell growth and proliferation, promoted cell cycle arrest, and induced cell senescence. We further revealed that SALL1 tumor suppressor activity depended on its ability to recruit NuRD and that this molecular process was controlled by MAPK p38 and ERK1/2, and mTOR signaling pathways in cancer cells. In addition, our complementary in vivo studies demonstrated that SALL1 expression and NuRD recruitment in breast tumor cells inhibited tumorigenesis and metastasis in breast cancer models in vivo. Collectively, these studies suggest that SALL1 functions as a tumor suppressor in breast cancer and directly controls cancer cell fate and metastasis.

## Results

### SALL1 expression is down-regulated in human breast cancer cell lines and tissues

**Fig. 1:**
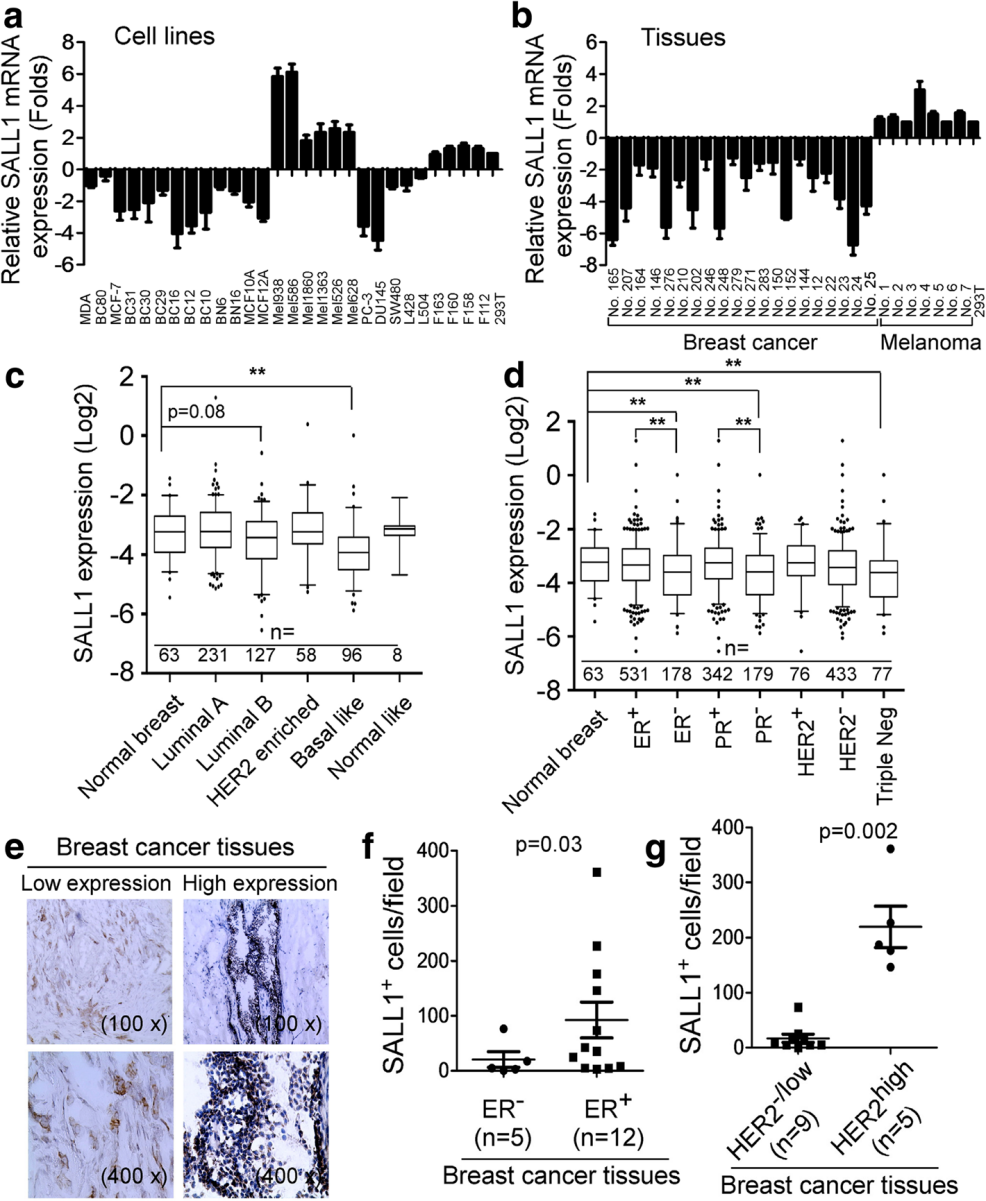
SALL1 expression is down-regulated in human breast cancer. **a** and **b** Gene expression levels of SALL1 in different cancer cell lines (in **a**) and in tumor tissues (in **b**) using Real-time PCR analyses. Tumor cell lines include breast cancer (human MDA-MB-231, MCF7, BC80, 31, 30, 29, 16, 12, and 10), melanoma (human Mel1938, Mel1586, Mel1860, Mel1363, Mel1526 and Mel1628), prostate cancer (PC3 and DU145), colon cancer (SW480), and lymphoma (L428 and L504). Normal breast cell lines (BN6, BN16, MCF10A and MCF12A), Fibroblasts (F163, F160, F158 and F112) and 293 T cells were included as controls. mRNA levels in each cell line and tissue were normalized to the relative quantity of GAPDH expression and then adjusted to SALL1 levels in 293 T cells (set as 1). Results shown in the histogram are mean ± SD from three independent experiments. **c** and **d** Association analyses of SALL1 expression with specific breast cancer subtypes. The data sets were accessed from the TCGA breast cancer Argilent microarray expression database downloaded from the cBioPortal (http://www.cbioportal.org/). The box plot indicated the log _2_ transformed mRNA median expression level of SALL1 in the tissues. N indicated the number of sample size of each tissue type. Mann-Whitney analysis was used to compare the SALL1 expression across the different breast cancer subtypes and normal tissues, and **p < 0.01 within the comparison groups. **e** SALL1 expression in tumor cells in breast cancer tissues was determined using the immunohistochemical staining. **f** and **g** SALL1 expression levels in breast cancer tissues with different ER and HER2 status. SALL1^+^ cell population in ER^+^ patients was significantly higher than that in ER^−^ patients. Furthermore, SALL1^+^ cell numbers in HER2^+^ patients were much higher than that in HER2^−^ patients. Tissue immunohistochemical staining and cell number counting were identical as in (**e**). Significance was determined by unpaired T test

We investigated whether SALL1 could function as a tumor suppressor and dissected the molecular mechanism by which it regulates human breast cancer. We first determined SALL1 gene expression levels in breast cancer cell lines using Real-time PCR analyses. In parallel, SALL1 expression in cancer cell lines from other types of cancers (melanoma, prostate cancer, colon cancer and lymphoma), as well as in normal breast primary cell lines, fibroblasts and 293 T cells were also determined. We observed markedly elevated SALL1 gene expression in all melanoma cell lines and moderate gene expression in normal fibroblasts and 293 T cells (Fig. 1a). In contrast, SALL1 gene expression levels in all the tumor cell lines from breast cancer, prostate cancer, colon cancer and lymphoma were significantly down-regulated. In addition, SALL1 expression in normal primary breast cells and cell lines (BN6, BN16, MCF10A and MCF12A) were also relatively low compared with normal fibroblasts and 293 T cells. These results were further confirmed in human breast and melanoma tumor tissues, showing down-regulation of SALL1 gene in breast cancer tissues (Fig. 1b). Clinically breast cancers can be classified into several distinct subtypes based on the histopathology and molecular characteristics which are associated with the therapeutic options and prognostic outcomes. To further investigate the association of SALL1 gene expression with breast cancer subtypes, we utilized The Cancer Genome Atlas (TCGA) normalized log_2_ transformed breast cancer agilent microarray expression data sets downloaded from the cBioPortal (http://www.cbioportal.org/) for our studies [27]. Breast cancers have been designated as luminal A, luminal B, epidermal growth factor receptor 2 (HER2) enriched, basal like and normal like, five important categories based on gene expression profiles [28]. We found a substantial decrease of SALL1 expression in the basal like breast cancer compared with that in normal breast tissue (*p* < 0.001) (Fig. 1c), which is consistent with the previous report [20]. Furthermore, we also compared SALL1 expression in breast cancer patients with different hormone receptors, estrogen receptor (ER) and Progesterone receptor (PR), or HER2 expression statuses (Fig. 1d). The analysis revealed that there was significantly lower expression of SALL1 in the ER^−^ breast cancer than that in the ER^+^ cancer tissues and normal breast tissues (*p* = 0.006 and *p* = 0.008, respectively). Similarly the SALL1 expression was lower in PR^−^ breast cancer than that in PR^+^ breast cancer or the normal breast tissues (*p* < 0.001 and *p* = 0.0059, respectively). Importantly, we also found a significant lower expression level of SALL1 in triple negative breast cancer tumors (ER^−^, PR^−^, and Her2^−^) compared with that in normal breast tissues (*p* = 0.0021). To further investigate the SALL1 expression in human breast cancer, we determined the SALL1^+^ cell numbers in cancer tissues from breast and melanoma cancer patients, using immunohistochemical staining analyses. Consistent with the gene expression results (Fig. 1b), we found that melanoma tumor tissues contained larger numbers of SALL1^+^ cells (mean 232/field), while in breast cancer tissues, the SALL1^+^ cells were low (mean 50/field) (Fig. 1e and Additional file 1: Figure S1A). Notably, SALL1^+^ cell numbers in ER^−^ patients were significant lower than those in ER^+^ patients (Fig. 1f). In addition, SALL1^+^ cell population in HER2^+^ patients was much higher than that in HER2^−^ patients (Fig. 1g). To further identify mechanism(s) responsible for the down-regulation of SALL1 gene in breast cancer, we explored the mutations and promoter methylation status of the SALL1 gene via The Catalogue of Somatic Mutations in Cancer (COSMIC) genome browser [27]. However, we did not identify any mutations that could account for SALL1 down-regulation in breast cancer (Additional file 1: Figure S1B). Previous study has shown that the SALL1 promoter was methylated in breast and other epithelial cancers [19]. We selected two probes that indicate a methylation difference of the SALL1 promoter in COSMIC between the primary tumor in breast cancer and solid normal tissues. We observed that the genes were highly methylated in these 2 regions of SALL1 promoter in the breast cancer tissues, which might be partially responsible for the down-regulation of SALL1 in breast cancer (Additional file 1: Figure S1C). Recent studies have shown that SALL4 is involved in leukemogenesis and a marker for hepatoblastoma and non-small cell lung carcinoma [15–17]. We therefore determined SALL4 gene expression in breast cancer cell lines and primary cancer tissues. In contrast to SALL1 gene expression, we found significantly higher expression of SALL4 in both breast cancer cell lines and primary cancer tissues, suggesting that SALL1 and SALL4 may have distinct functional roles in the regulation of breast cancer (Additional file 1: Figure S1D and E). Collectively, our results suggest that SALL1 expression is significantly down-regulated in human breast cancer cells and in certain types of breast cancer tissues (especially in triple negative breast cancer), which may play a critical role in the pathogenesis of human breast cancer.

### SALL1 over-expression in breast cancer cells inhibits tumor cell growth and proliferation, and promotes cell cycle arrest

**Fig. 2:**
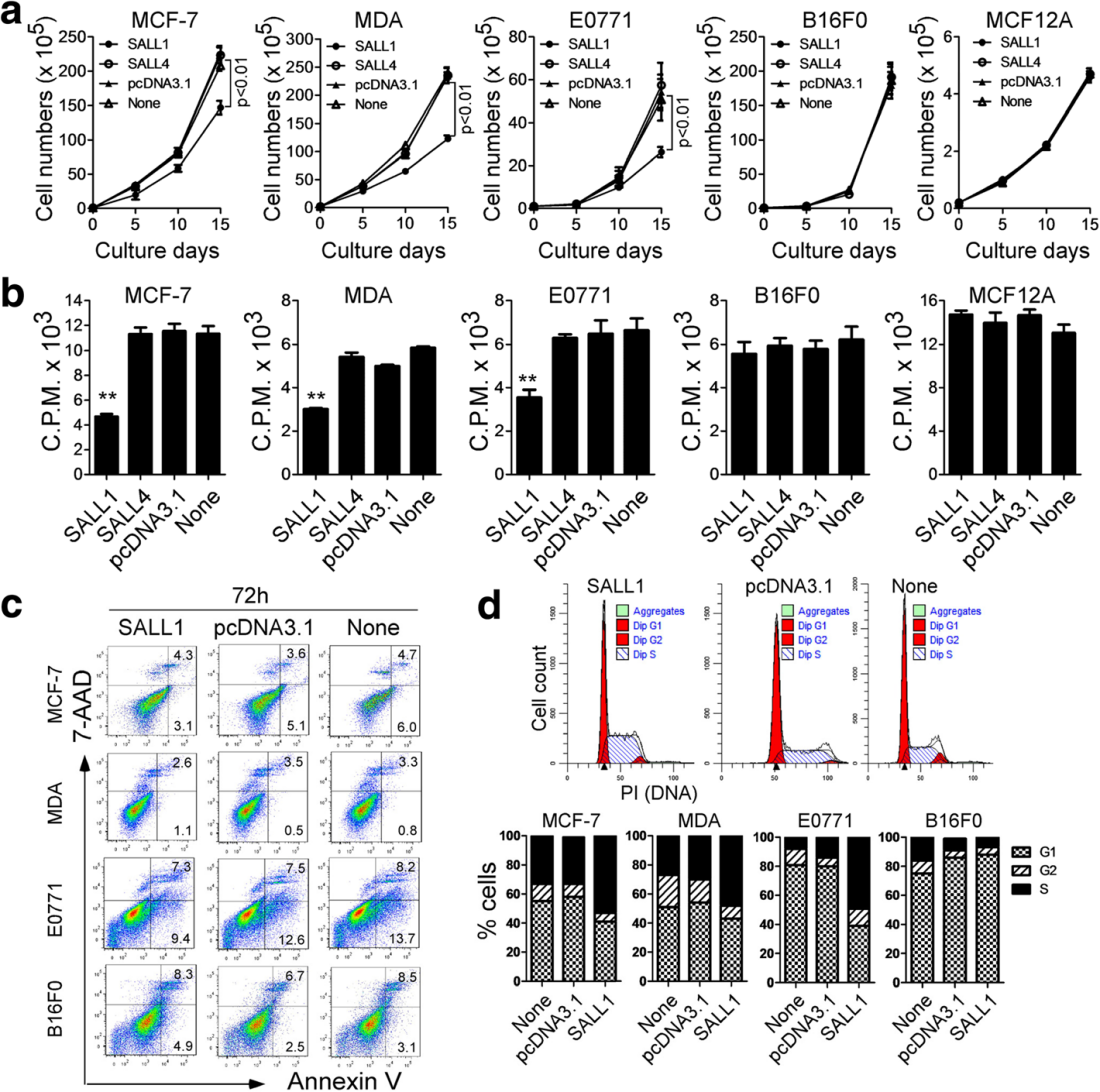
Over-expression of SALL1 in breast cancer cells inhibits tumor cell growth and proliferation, and promotes cell cycle arrest. **a** and **b** Transfection of SALL1 but not SALL4 significantly inhibited breast cancer cell growth and proliferation. However, over-expression of SALL1 in B16F0 melanoma cells and normal MCF12A cells (controls) did not affect cell growth and proliferation. Cells transfected with or without plasmids pcDNA3.1-SALL1, pcDNA3.1-SALL4, and pcDNA3.1, were cultured at a starting number of 2 × 10^4^/well in 24 wells (**a**), or 1 × 10^4^/well in 96-well plates (**b**). Cell growth was evaluated at different time points using by counting cell number (in **a**), and cell proliferation was determined using [^3^H]-thymidine assays (in **b**). Data are mean ± SD from three independent experiments with similar results. **p < 0.01 compared with the vector control group. **c** The suppression of breast cancer cell proliferation and growth mediated by SALL1 expression is not due to cell apoptosis. Transfected breast cancer and melanoma cells were cultured for additional 72 h. Apoptosis in transfected tumor cells was analyzed after staining with PE-labeled Annexin V and 7-AAD. Data shown are representative of three independent experiments with similar results. **d** SALL1 transfection in MCF-7, MDA-MB-231 and E0771 cells, but not in melanoma B16F0 cells significantly promoted cancer cell cycle arrest in S phase and decrease in G0/G1 phase. Cell treatment was the same as in (**c**). Cell cycle distribution in tumor cells was analyzed after incubation with 10 μg/ml propidium iodide and 100 μg/ml RNase A. B16F0 melanoma cells served as a control. Data are representative of three independent experiments with similar results

To test whether SALL1 is a tumor suppressor [20], we determined the effect on cancer cell growth and function by SALL1. SALL1 was transfected into human and murine breast cancer cell lines MDA-MB-231 (TNBC), MCF7 and E0771 (basal-like) (all with no or minor expression of SALL1) for the gain-of-function studies. Prostate cancer cell line PC-3 (low SALL1 expression) and melanoma cell line B16F0 (high SALL1 expression), as well as normal breast cell line MCF12A cells were included as controls. Tumor cell growth and proliferation were determined by analyzing cell growth curves and [^3^H]-thymidine incorporation assays. We found that transfection of SALL1, but not SALL4 or vector in MCF-7, MDA-MB-231, E0771 and PC-3 tumor cells significantly inhibited cell growth and proliferation. However, over-expression of SALL1 in B16F0 melanoma cells, and normal breast cell MCF12A did not affect cell growth and proliferation (Fig. 2a and b, and Additional file 1: Figure S2A and B). These results further suggest that SALL1 directly inhibits breast cancer cell growth, but it may have different functions in the other types of cancers.

Suppression of tumor cell proliferation and growth mediated by SALL1 expression could be due to the induction of apoptosis or cytolysis in the tumor cells. We therefore measured apoptosis and cell death in SALL1-transfeced breast tumor cells. We found that breast tumor cells MCF7, MDA-MB-231 and E0771 in medium alone or transfected with control vector contained some apoptotic cells (around 10% in MCF-7, 4% in MDA, and 20% in E0771). However, overexpression of SALL1 in cancer cells did not induce increased apoptosis or cell death in breast cancer cell lines (Fig. 2c and Additional file 1: Figure S2C). In parallel, we studied the cell cycle distribution of the breast cancer cells transfected with SALL1. SALL1 transfection in MCF-7, MDA and E0771 cells significantly induced cancer cells to arrest in S phase and decrease in G0/G1 phase (Fig. 2d). Notably, transfection of SALL1 in melanoma B16F0 cells induced neither cell apoptosis nor cell cycle arrest. To further identify the potential mechanism responsible for the SALL1-mediated breast cancer cell arrest in S phase, we determined the cell cycle regulation gene expressions in MDA-MB-231 cells using Real-time PCR analysis, including Cyclin A2, B1, D1 and E1, as well as CDK2, 4 and 6. We observed that transfection with SALL1 significantly increased the gene expressions of Cyclin A2, Cyclin B1, Cyclin E1, CDK2 and CDK4 in breast cancer MDA cells, which are important for checkpoint regulation in G1-S transition and S phases (Additional file 1: Figure S3). These data suggest that over-expression of SALL1 in breast cancer strongly suppresses tumor growth and proliferation, as well as induces cell cycle arrest, which is mechanistically independent of apoptosis or cytolysis in tumor cells.

### Knockdown of SALL1 in breast cancer cells promotes tumor cell growth, proliferation, and colony formation

**Fig. 3:**
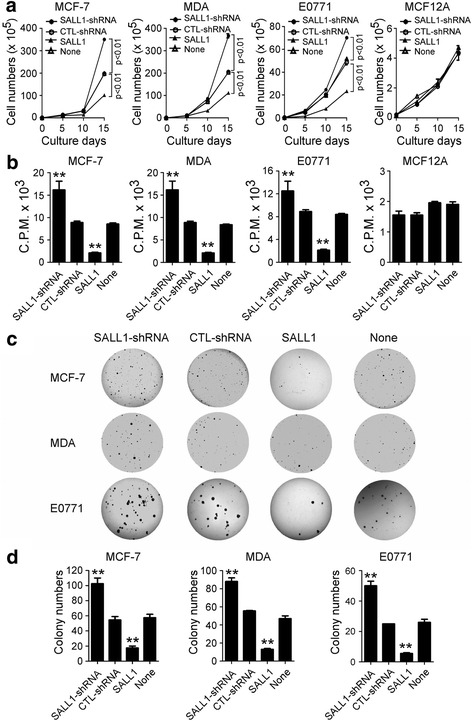
Knockdown of SALL1 in breast cancer cells promotes tumor cell growth, proliferation, and colony formation. **a** and **b** Knockdown of SALL1 significantly promoted breast cancer cell growth and proliferation. However, over-expression of SALL1 dramatically inhibited breast cancer cell growth and proliferation. In addition, knockdown or over-expression of SALL1 in normal breast cells MCF12A (control) did not affect cell growth and proliferation. Tumor or normal cells were infected with lentivirus carrying shRNA specific for SALL1 or scramble shRNA control, or transfected with or without plasmids pcDNA3.1-SALL1, and then were cultured at a starting number of 2 × 10^4^/well in 24 wells (**a**), or 1 × 10^4^/well in 96-well plates (**b**). Cell growth was evaluated at different time points using by counting cell number (in **a**), and cell proliferation was determined using [^3^H]-thymidine assays (in **b**). Data are mean ± SD from three independent experiments with similar results. **p < 0.01 compared with the control shRNA and medium only groups. **c** and **d** Knockdown of SALL1 expression in breast cancer cells dramatically increased numbers and sizes of tumor colonies, while over-expression of SALL1 significantly inhibited breast cancer cell colony formation ability. Five thousand per well of tumor cells with the indicated treatments were cultured in soft agar in 6-well plate for 3 weeks and examined for their anchorage-dependent colony formation ability. Results shown in the histogram (in **d**) are mean ± SD from three independent experiments. **p < 0.01 compared with the control shRNA and medium only groups

To further confirm the functional role of SALL1 in regulating breast cancer cell growth, we also utilized a loss-of-function strategy to knockdown SALL1 gene expression with lentivirus-based shRNA in breast cancer cell lines and then determined its effect on tumor growth and proliferation. As expected, silencing of SALL1 expression in MCF-7, MDA-MB-231 and E0771 breast cancer cells dramatically promoted tumor growth and increased cell proliferation compared with lentivirus carrying control shRNA infected breast cancer cells (Fig. 3a and b). Furthermore, the numbers and sizes of tumor cell colonies were also significantly increased in three breast tumor cell lines after knockdown of SALL1 in a colony formation assay (Fig. 3c and d). In contrast, over-expression of SALL1 in breast cancer cells dramatically inhibited tumor cell grown and proliferation, as well as decreased colony numbers, which were consistent with the results shown in Fig. 2. In addition, we did not observe any effects of SALL1 overexpression or down-regulation on cell growth and proliferation in normal breast MCF12A cells. These results further suggest that SALL1 expression in breast cancer cells directly controlled cell growth and function.

### SALL1 over-expression in breast cancer cells induces tumor cell senescence

**Fig. 4:**
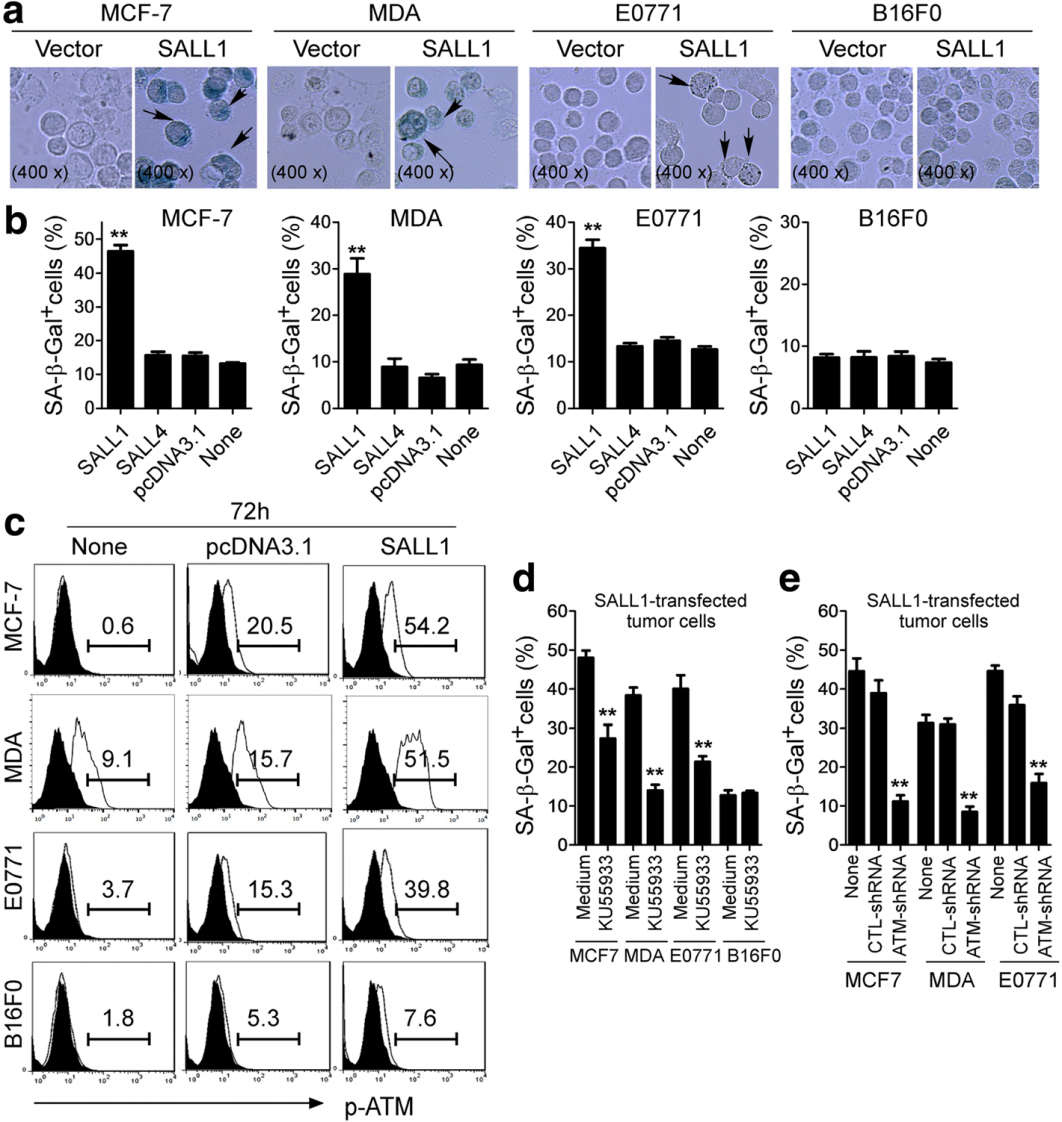
SALL1 over-expression in breast cancer cells induces tumor cell senescence and ATM-associated DNA damage response. **a** and **b** Transfection of SALL1, but not SALL4 in MCF-7, MDA-MB-231 and E0771 breast cancer cells significantly induced the increased SA-β-Gal^+^ cells. In contrast, over-expression of SALL1 in B16F0 melanoma cells did not increase senescent cell populations. Transfected tumor cells were cultured for an additional 5 days. Senescent cells were analyzed using the SA-β-Gal activity assay and the SA-β-Gal^+^ tumor cells were identified with dark blue granules as indicated by the arrows (in **a**). Data shown in (**b**) are mean ± SD from three independent experiments with similar results. **p < 0.01 compared with the vector control group. **c** SALL1 expression in breast cancer cells induced phosphorylated activation of ATM in the transfected cells. Transfected tumor cells were determined for the p-ATM expression after culture for 3 additional days using FACS analyses. **d** Pretreatment of breast cancer cells with an ATM specific inhibitor KU55933 significantly prevented the induction of tumor cell senescence induced by SALL1 expression. Tumor cells were pretreated with or without KU55933 (20 μM) for 1 day, and then transfected with SALL1. SA-β-Gal expression in the transfected tumor cells was determined with SA-β-Gal staining after culture for 3 additional days. Data shown are mean ± SD from three independent experiments, and paired t-test was performed. **p < 0.01, compared with the medium only control group. **e** Knockdown of ATM gene by shRNAs in MCF-7, MDA-MB-231 and E0771 cells dramatically blocked SALL1-induced tumor cell senescence. Breast cancer cells were transfected with lenti-shRNAs specific for ATM or control shRNAs. Transduced cancer cells were then transfected with SALL1 and cultured for 5 days. The SA-β-Gal^+^ cancer cells were determined. **p < 0.01, compared with the group transduced with the control shRNA. Data shown are representative of three independent experiments with similar results

Senescent human cells have permanent growth arrest, which could occur due to telomere shortening or a DNA damage response [29, 30]. We therefore reasoned that induction of senescence might be the mechanism involved in the suppressed cell growth and proliferation mediated by SALL1 overexpression in breast cancer cells. In addition to cell cycle arrest and morphologic characteristics, SA-β-Gal is the first biomarker used to identify senescent human cells [31, 32]. We observed that transfection of SALL1 in MCF-7, MDA, and E0771 tumor cells significantly increased the number of SA-β-Gal^+^ cells, indicating the induction of tumor cell senescence (Fig. 4a and b, and Additional file 1: Figure S4A). In contrast, transfection with *SALL4* or control vector in these tumor cells did not induce increased SA-β-Gal expression. In addition, we did not observe increased senescent cell populations in melanoma B16F0 cells after over-expression of SALL1.

The induction of DNA damage is the key molecular process in senescent cells, which could be induced by telomere erosion and/or other forms of stress. The nuclear kinase ataxia-telangiectasia mutated protein (ATM) is the chief inducer of the DNA-damage response. We thus determined whether induction of ATM-associated DNA damage is the main trigger for SALL1-induced senescence in breast tumor cells [33, 34]. Over-expression of SALL1 significantly induced active, phosphorylated ATM in MCF-7, MDA and E0771 cancer cells (Fig. 4c and Additional file 1: Figure S4B). In addition, we further investigated the other key DNA damage response proteins involved in the induction of senescence due to the DNA damage response. These proteins include ATM substrates H2AX and 53BP1, as well as the downstream target checkpoint kinase 2 (CHK2) [30, 34]. We observed that transfection of SALL1, but not SALL4 or control vector also significantly induced phosphorylation of H2AX, 53BP1 and CHK2 in MCF-7, MDA and E0771 cells (Data not shown). To confirm the involvement of ATM-associated DNA damage response in SALL1-mediated breast cancer cell senescence, we next determined whether we can prevent the SALL1-mediated senescence in breast cancer cells through the functional blockade of ATM-induced DNA damage using loss-of-function approaches with the specific pharmacological ATM inhibitor KU55933 and shRNA against ATM. As shown in Fig. 4d, treatment of MCF-7, MDA and E0771 breast cancer cells with KU55933 dramatically suppressed the phosphorylation of ATM in SALL1-transfected tumor cells and prevented induction of senescence in tumor cells. In addition, knockdown of ATM expression with shRNA significantly decreased the senescent cell populations in SALL1-transfected breast tumor cells, further confirming the involvement of the ATM-associated DNA damage response in SALL1-induced tumor cell senescence (Fig. 4e). These data provide the first evidence that suppression of breast cancer growth and proliferation mediated by SALL1 expression is due to the induction of tumor cell senescence.

### SALL1 recruits NuRD in breast cancer performing a tumor suppressor function

**Fig. 5:**
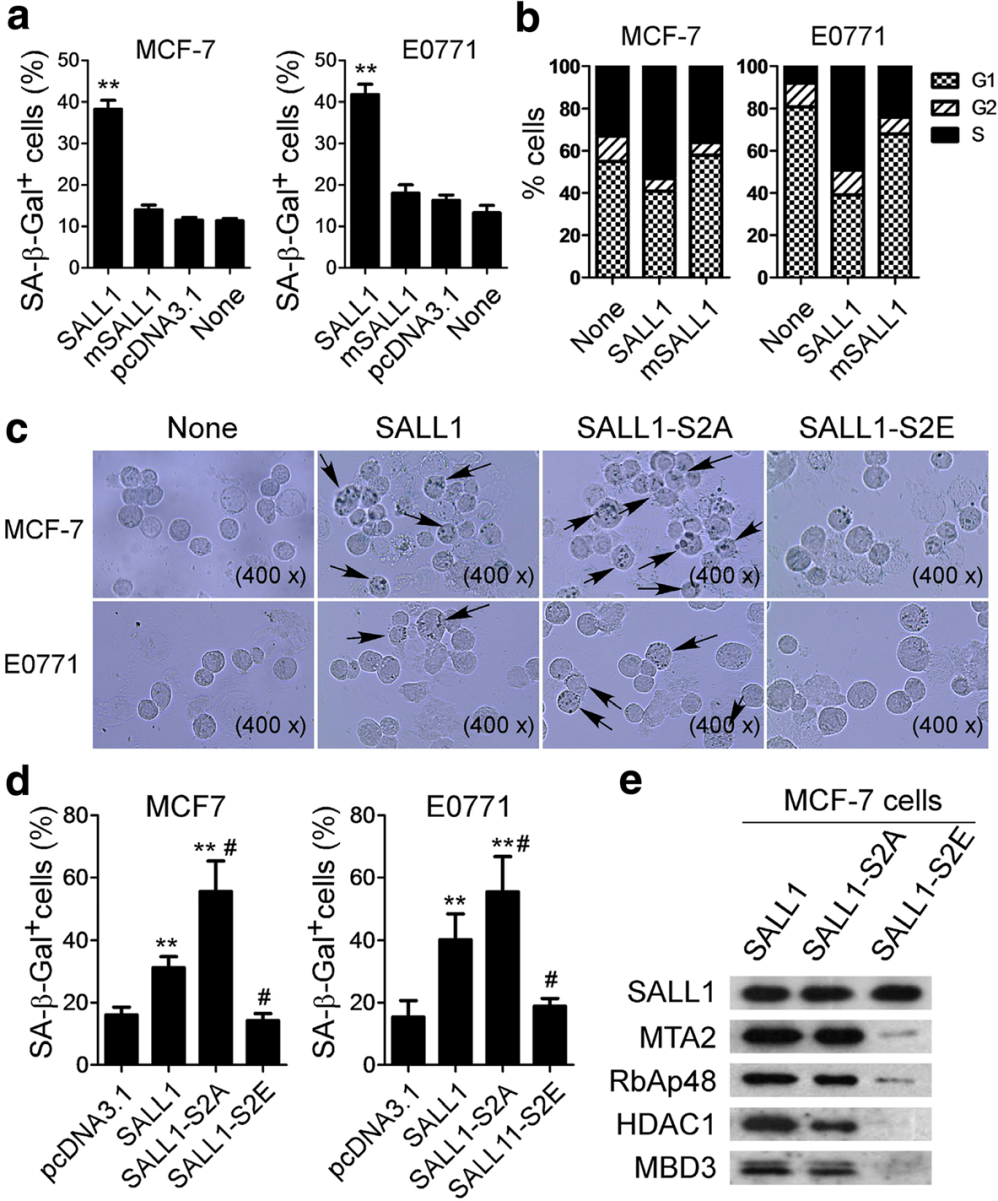
Involvement of NuRD complex in the regulation of breast cancer cell senescence and suppression mediated by SALL1. **a** and **b** Transfection of mutated SALL1 (mSALL1, deleted the NuRD binding peptide motif of conserved 12-amino) in MCF-7 and E0771 cancer cells did not induce SA-β-Gal^+^ cell populations (in **a**) and promote cancer cell cycle arrest in S phase (in **b**). In contrast, transfection of full-length SALL1 into MCF-7 and E0771 breast cancer cells significantly induced tumor cell senescence (around 40%) and promoted cell cycle arrest in S phase. Breast cancer cells were transfected with the indicated constructs and cultured for additional 72 h. Senescent cells were analyzed using the SA-β-Gal activity assay and the cell cycle distribution in tumor cells was analyzed after incubation with propidium iodide. Data shown in (**a**) are mean ± SD from three independent experiments with similar results. **p < 0.01 compared with the mSALL1 and vector control groups. **c** and **d** Transfection of SALL1-S2E into MCF-7 and E0771 breast cancer cells lost the ability to induce tumor cell senescence. However, transfection of SALL1-S2A into breast cancer cells significantly augmented senescence induction in both cell lines compared with that of in wild type SALL1-transfected tumor cells. Cell transfection procedure and SA-β-Gal^+^ cell determination were identical to (**a**). SALL1-S2E: substitution of the serine with a glutamic acid in SALL1. SALL1-S2A: mutating the serine to an alanine in SALL1. SA-β-Gal^+^ tumor cells were identified with dark blue granules as indicated by the arrows (in **c**). Data shown in (**d**) are mean ± SD from three independent experiments with similar results. **p < 0.01, compared with the vector control group. #*p* < 0.01, compared with the wild type SALL1 group. **e** Transfection of wild type SALL1 and SALL1-S2A into MCF-7 tumor cells recruited NuRD complex components determined with GST pulldown analyses. In contrast, transfection of SALL1-S2E markedly disrupted recruitment of NuRD components. MCF-7 cells were transfected with or without plasmids pEBG-SALL1, pEBG-SALL1-S2A, and pEBG-SALL1-S2E, and cultured for 3 days. Total protein lysates precipitated with Protein G-Sepharose beads. Pulldowns were analyzed by western blotting with antibodies against SALL1, HDAC1, MTA2, MBD3 and RbAp46/48

Our previous studies have shown that endogenous SALL1 binds to the NuRD complex to regulate gene transcription and specific developmental processes [4, 6, 21–23]. We further identified a highly conserved 12-amino acid motif in the SALL1 protein that is sufficient for the recruitment of NuRD [22]. Importantly, increasing evidence suggests that the NuRD complex plays an essential role in regulating oncogenesis and metastasis programs of breast cancer [24–26]. We therefore hypothesized that SALL1-mediated suppression of breast cancer growth and proliferation, and induction of tumor cell senescence may also act through the recruitment of the NuRD complex. To test this hypothesis, we first transfected a mutated SALL1 (mSALL1) encoding a protein in which the conserved 12-amino acid peptide motif that specifically binds to NuRD was deleted, into breast cancer cells and determined the capacity for senescence induction (Additional file 1: Figure S5). Consistent with the above results, transfection of full-length SALL1 into MCF-7 and E0771 breast cancer cells significantly induced tumor cell senescence (around 40%) and promoted cell cycle arrest in S phase in 3 days (Fig. 5a and b). However, transfection of the mutated SALL1 in the breast cancer cells did not have any effects on cell senescence and cell cycle, similar to that of control vector, suggesting that breast cancer cell senescence mediated by SALL1 depends on NuRD recruitment.

In our efforts to identify the relationship and direct interactions between SALL1 and NuRD, we have demonstrated that protein kinase C phosphorylates serine 2 of the SALL1 repression motif and regulates the association with NuRD [21]. Furthermore, we showed that substitution of the serine with a glutamic acid (SALL1-S2E, phosphomimetic) significantly abolished the effect on NuRD recruitment and repression activity; whereas mutating the serine to an alanine (SALL1-S2A) modestly increased the transcriptional repression [21, 22] (Additional file 1: Figure S5). We next utilized SALL1 constructs with these two separation-of-function mutations to test its effects on senescence induction in breast cancer cells. As we expected, transfection of SALL1-S2E into MCF-7 and E0771 breast cancer cells lost the ability to induce tumor cell senescence. In contrast, transfection of SALL1-S2A into tumor cells significantly augmented senescence induction in both cell lines compared with that of wild type SALL1-transfected tumor cells (Fig. 5c and d). To confirm the physical interaction of SALL1 with the NuRD complex in cancer cells, we transfected MCF-7 breast cancer cells with GST fusions of wild type SALL1, or SALL1-S2A and SALL1-S2E. GST-SALL1 fusion proteins were isolated on glutathione-Sepharose beads. Western-blot assays were then performed to determine the expression of components of the NuRD complex, including MTA2, RbAp46/48, HDAC1 and MBD3, after SALL1 and GST pulldown [21, 22]. Transfection of the three fusion constructs equivalently expressed SALL1 protein (Fig. 5e). Expression of wild type SALL1 and SALL1-S2A in MCF-7 tumor cells recruited the endogenous NuRD complex components. However, GST-SALL-S2E did not pulldown NuRD components even though it is expressed at a level comparable to wild type SALL1. Collectively, these results clearly indicate that SALL1 recruits NuRD in breast cancer cells resulting in suppression of tumor growth and proliferation, and induction of tumor cell senescence.

### SALL1 induces selective modulation of MAPK p38 and ERK1/2, and mTOR signaling pathways in breast cancer cells

SALL proteins heterodimerize and in some contexts can cross-regulate their respective expression. We thus determined whether SALL1 could alter the expression of other members in the SALL family mediating breast cancer growth suppression [10]. Our results showed that transfection of SALL1 in both MCF-7 and E0771 cells did not change the gene expression levels of SALL2, SALL3 and SALL4 at different time points using Real-time PCR analyses (Additional file 1: Figure S6).

**Fig. 6:**
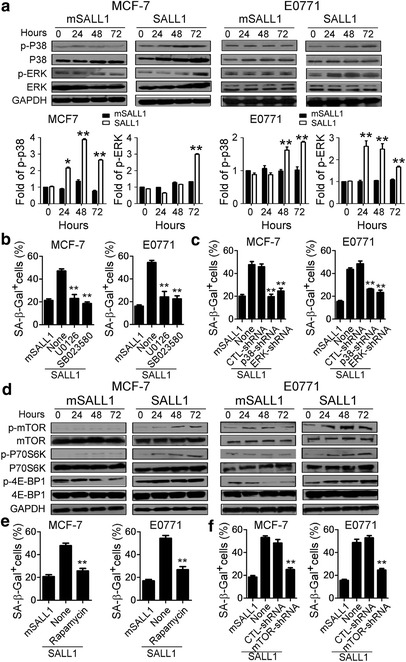
MAPK p38 and ERK1/2, and mTOR signaling pathways control the molecular process of SALL1-induced breast cancer cell senescence. **a** Transfection of wild type SALL1 but not mutated SALL1 in MCF-7 and E0771 cells induced phosphorylation of ERK and p38 in senescent tumor cells. Transfected breast cancer cells were cultured for different time points and cell lysates were prepared for western blot analyses. Results from western blot analyses of phosphorylated activation of ERK and p38 were shown in the upper panel. Phosphorylated ERK and p38 protein levels shown in the lower histogram were analyzed quantitatively and compared against the GAPDH expression level with a densitometer. Results shown in the histogram were means±SD from 3 independent experiments. *p<0.05 and **p<0.01 compared with the mutated SALL1 transfected group. **b** Inhibition of ERK1/2 or p38 signaling pathways by specific pharmacological inhibitors significantly prevented breast cancer cell senescence induced by SALL1, resulting in decreased SA-β-Gal expression. SALL1-transfected MCF-7 and E0771 cancer cells were cultured in the presence or absence of inhibitors U0126 or SB203580 (10μM) for 5 days. The treated tumor cells were analyzed for SA-β-Gal expression. Data shown are mean ± SD from three independent experiments with similar results. **p < 0.01, compared with the SALL1-transfected group but not treated with inhibitor. **c** Knockdown of ERK1/2 and p38 genes by shRNAs in MCF-7 and E0771 cells dramatically blocked SALL1-induced tumor cell senescence. Breast cancer cells were infected with lenti-shRNAs specific for ERK1/2 or p38 molecules. Transduced (GFP^+^) cancer cells were purified by FACS sorting and then transfected with SALL1 and cultured for 5 days. The SA-β-Gal^+^ cancer cells were determined. **p < 0.01, compared with the group transduced with the control shRNA. Data shown are representative of three independent experiments with similar results. **d** Phosphorylation and subsequent activation of mTOR signaling in breast cancer cells transfected with SALL1 but not mSALL1. Transfected MCF-7 and E0771 cells cultured for different times, and then were collected for western blot analyses of total and phosphorylated mTOR, p70S6K, and 4E-BP1 protein levels. **e** Rapamycin markedly inhibited the SALL1-mediated breast cancer cell senescence. SALL1-transfected MCF-7 and E0771 cancer cells were cultured in the presence or absence of mTOR inhibitor rapamycin (5 μM) for 5 days. The treated tumor cells were stained for SA-β-Gal expression. Data shown are mean ± SD from three independent experiments with similar results. **p < 0.01, compared with the SALL1-transfected group but not treated with inhibitor. **f** Knockdown of mTOR by shRNA in MCF-7 and E0771 cells significantly blocked SALL1-induced tumor cell senescence. Transfection procedure was identical to (**c**) and SA-β-Gal^+^ cancer cells were determined. **p < 0.01, compared with the group transduced with the control shRNA. Data shown are representative of three independent experiments with similar results

MAPK signaling pathways play a major role in regulating cell cycle re-entry and oncogenic ras-induced senescence [35]. It has been reported that ERK1/2 and p38 activation can induce p21-dependent G1 cell cycle arrest [36]. Our recent studies further demonstrated that MAPK ERK1/2 and p38 signaling controls the molecular process of human CD4^+^CD25^hi^FoxP3^+^ Treg-induced responder T cell senescence [32, 34]. We therefore explored whether SALL1-induced breast cancer cell cycle S phase arrest and conversion of cancer cells into senescent cells involved MAPK signaling modulation. We first determined the activation and phosphorylation of MAPKs, including ERK1/2, p38 and JNK in breast cancer cells transfected with SALL1 using western blot analyses. We found that transfection of SALL1 but not mutated SALL1 selectively activated ERK1/2 and p38, but not JNK, resulting in significantly enhanced phosphorylation of ERK1/2 and p38 in both MCF-7 and E0771 breast cancer cells (Fig. 6a). To further determine the role of ERK1/2 and p38 signaling in controlling the molecular process of SALL1-induced senescence in breast cancer cells, we utilized loss-of-function strategies with specific pharmacological inhibitors and lentivirus-based shRNAs to block ERK1/2 and p38 activities in breast cancer cells, as we previously described [32]. The optimal concentrations (10 μM) of different inhibitors used for our experiments, including SB203580 (p38 inhibitor) and U0126 (ERK1/2 inhibitor), were selected based on their toxic effects on tumor cell viability and proliferation. As shown in Fig. 6b, we observed that inhibitors U0126 and SB203580 significantly reduced SALL1-induced senescent cell populations in both MCF-7 and E0771 breast tumor cells. We then used shRNAs to specifically knock down p38 and ERK1/2 genes in MCF-7 and E0771 cells, and measured the effects on SALL1-induced tumor cell senescence. Consistent with the results obtained in inhibitor experiments described above, knockdown of p38 and ERK1/2 in MCF-7 and E0771 cells significantly decreased the senescence induction in SALL1-transfected tumor cells (Fig. 6c). These results suggest that SALL1 expression in breast cancer cells induces selective modulation of specific MAPK p38 and ERK1/2 signaling pathways in tumor cells that control the molecular process of SALL1-induced tumor cell senescence and growth suppression. Moreover, this process depends on an intact NuRD-recruitment motif in SALL1.

In addition to MAPK signaling, mTOR kinase signaling activation is important for tumor cell proliferation and senescence induction [37–41]. We next investigated whether mTOR signaling is also involved in the SALL1-induced breast cancer growth inhibition and senescence induction. We determined the activation of mTOR and its downstream substrates p70S6K and 4E-BP1, in breast tumor cells after transfection with SALL1 [42]. Transfection of SALL1 but not mutated SALL1 in both MCF-7 and E0771 breast tumor cells significantly induced the phosphorylation of mTOR, p70S6K, and 4E-BP1, further confirming the activation of mTOR signaling in tumor cells after SALL1 expression (Fig. 6d). Using loss-of-function strategies, we demonstrated that the mTOR inhibitor rapamycin and shRNA to specifically knock down mTOR gene expression in breast cancer cells dramatically prevented induction of senescence in tumor cells mediated by SALL1 expression (Fig. 6e and f). These results suggest that the mTOR signaling pathway is also critical in regulating breast cancer cell senescence mediated by SALL1 expression. Furthermore, our studies indicate that SALL1 expression in breast cancer selectively utilizes both MAPK and mTOR signaling pathways controlling tumor cell fate and functions.

### SALL1 expression in breast cancer cells inhibits tumorigenesis and metastasis in vivo

**Fig. 7:**
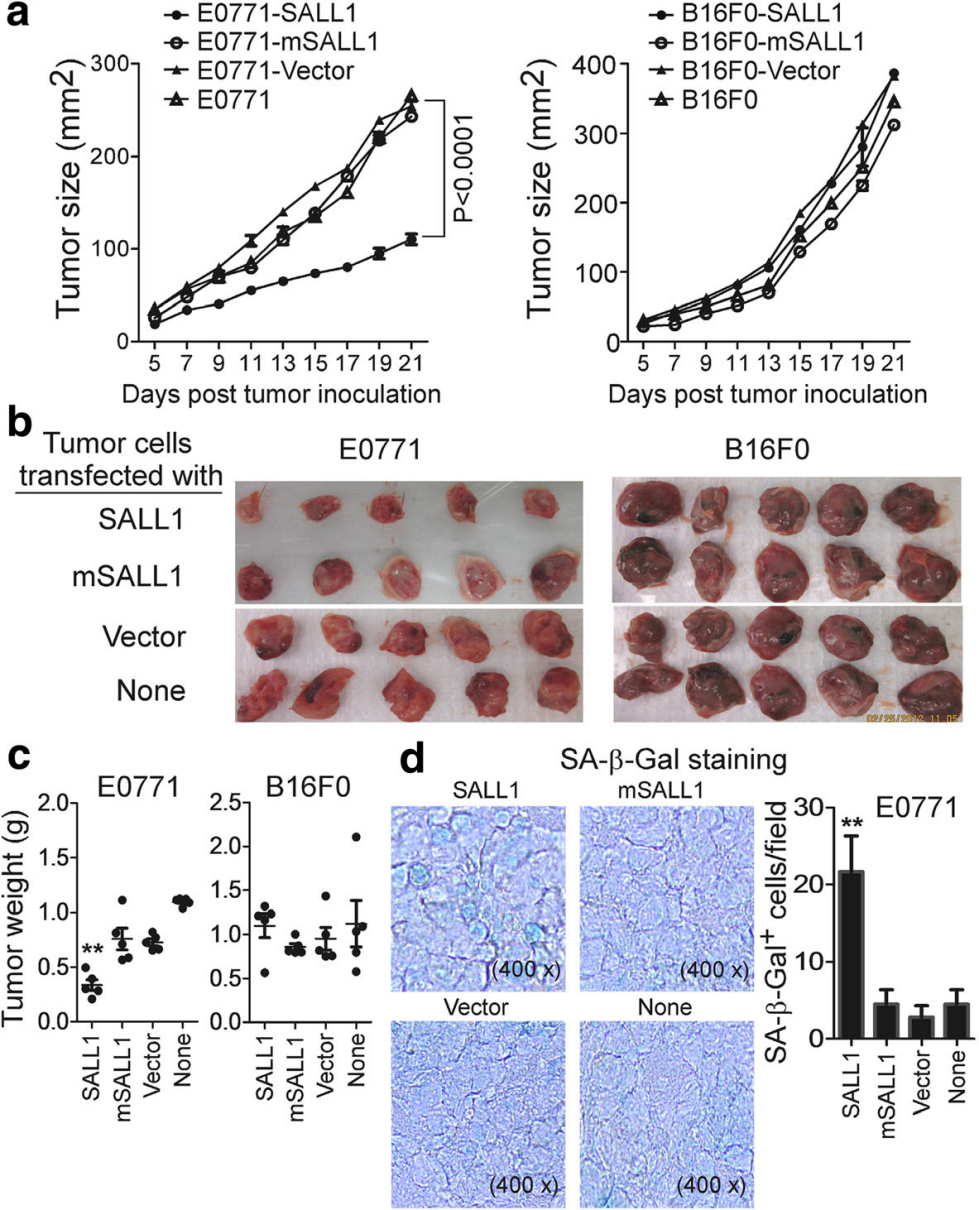
SALL1 over-expression in breast cancer cells inhibited tumor growth and development in vivo. **a** Over-expression of SALL1 in E0771 breast cancer cells dramatically inhibited tumor growth in NSG immunodeficient mice. However, SALL1 over-expression in B16F0 melanoma cells did not affect tumor development. E0771 (2 × 10^5^/mouse) and B16F0 (1 × 10^5^/mouse) tumor cells infected with lentivirus carrying SALL1, mSALL1 or vector, were subcutaneously injected into NSG mice. Tumor volumes were measured and presented as mean ± SD (*n* = 5 mice per group). *P* values were determined by the one-way analysis of variance (ANOVA). Similar results were obtained in three repeated experiments. **b** Representative image of the xenograft tumors obtained from the indicated groups at the endpoint of the experiments (day 21). **c** E0771 breast cancer cells over-expressed SALL1 had much lower tumor weight compared with that of other groups. Furthermore, SALL1 over-expression in B16F0 melanoma cells did not affect tumor weight. Results shown are mean ± SD of the xenograft tumor weights from the indicated groups in the two models at the endpoint of the experiments (day 21) (*n* = 5 mice per group). **p < 0.01, compared with the control groups transfected with mSALL1 and vector using unpaired t-test. **d** Large amounts of senescent tumor cells were observed in SALL1-transfected E0771 tumor tissues in NSG mice. SA-β-Gal expression was determined in the tumor frozen tissues from different groups at the endpoint of experiment. Left panels are photomicrographs of SA-β-Gal expression in tumor tissues from different groups. Right panel is the mean ± SD of SA-β-Gal^+^ cell numbers per high microscope field (× 400) in the tumor tissues from 5 mice of each group. **p < 0.01, compared with the control mice of mSALL1 and vector groups using unpaired t-test

These in vitro studies provided us with important information regarding the mechanism and molecular signaling of SALL1 in suppressing breast cancer tumor cell growth and metastasis. We next performed complementary in vivo studies, using murine breast cancer E0771 cells in humanized NOD-scid IL2Rγ^null^ (NSG) mouse models, and explored whether SALL1 functions as a tumor suppressor for the tumorigenesis and metastasis of breast cancer in vivo. The E0771 cell line is a basal-like and derived from medullary breast adenocarcinoma cells, representing a good model for spontaneously developed breast cancer [43, 44]. We also included murine B16F0 melanoma cells as a tumor control for this study. We first performed xenograft models to investigate whether over-expression of SALL1 in breast cancer cells can affect tumor growth and development. Mouse E0771 and B16F0 tumor cells infected with Lentivirus carrying SALL1, mSALL1 or vector, were subcutaneously injected into NSG mice. Tumor growth was evaluated. At the end of the experiments, tumors were isolated from the sacrificed mice and weighed. As shown in Fig. 7a, we observed that E0771 tumor cells alone or transfected with mutated SALL1 gene or vector control, grew progressively in NSG mice. However, over-expression of wild type SALL1 in E0771 cells dramatically inhibited breast tumor progression and growth. Furthermore, tumor sizes collected from the E0771-SALL1 group on day 21 post inoculation were significantly smaller than those in the control groups of E0771 tumor alone, E0771 cells transfected with mSALL1 or vector (Fig. 7b). In addition, the average tumor weights obtained from the E0771-SALL1 group were much lower than those of the three control groups (Fig. 7c). Notably, tumor growth, tumor sizes and weights were very similar among the three control groups of E0771 tumor alone, E0771 cells transfected with mSALL1 or vector. In contrast to the E0771 breast tumor model, there were no differences of tumor growth, progression and sizes among the experimental groups of B16F0 cells transfected with SALL1, mSALL1 or vector, in the B16F0 melanoma model (Fig. 7a-c). These results were consistent with our in vitro studies showing that SALL1 had different effects and functions in breast and melanoma tumor cells. Furthermore, we confirmed, using histochemical staining of SA-β-Gal expression on sections from embedded tumor tissues, that a high amount of senescent tumor cells were observed in tumor tissues obtained from the E0771-SALL1 group, but not from the control groups of E0771 tumor alone, E0771 cells transfected with mSALL1 or vector (Fig. 7d). These results clearly suggest that SALL1 expression in breast tumor cells directly controls tumor growth and tumorigenesis.

**Fig. 8:**
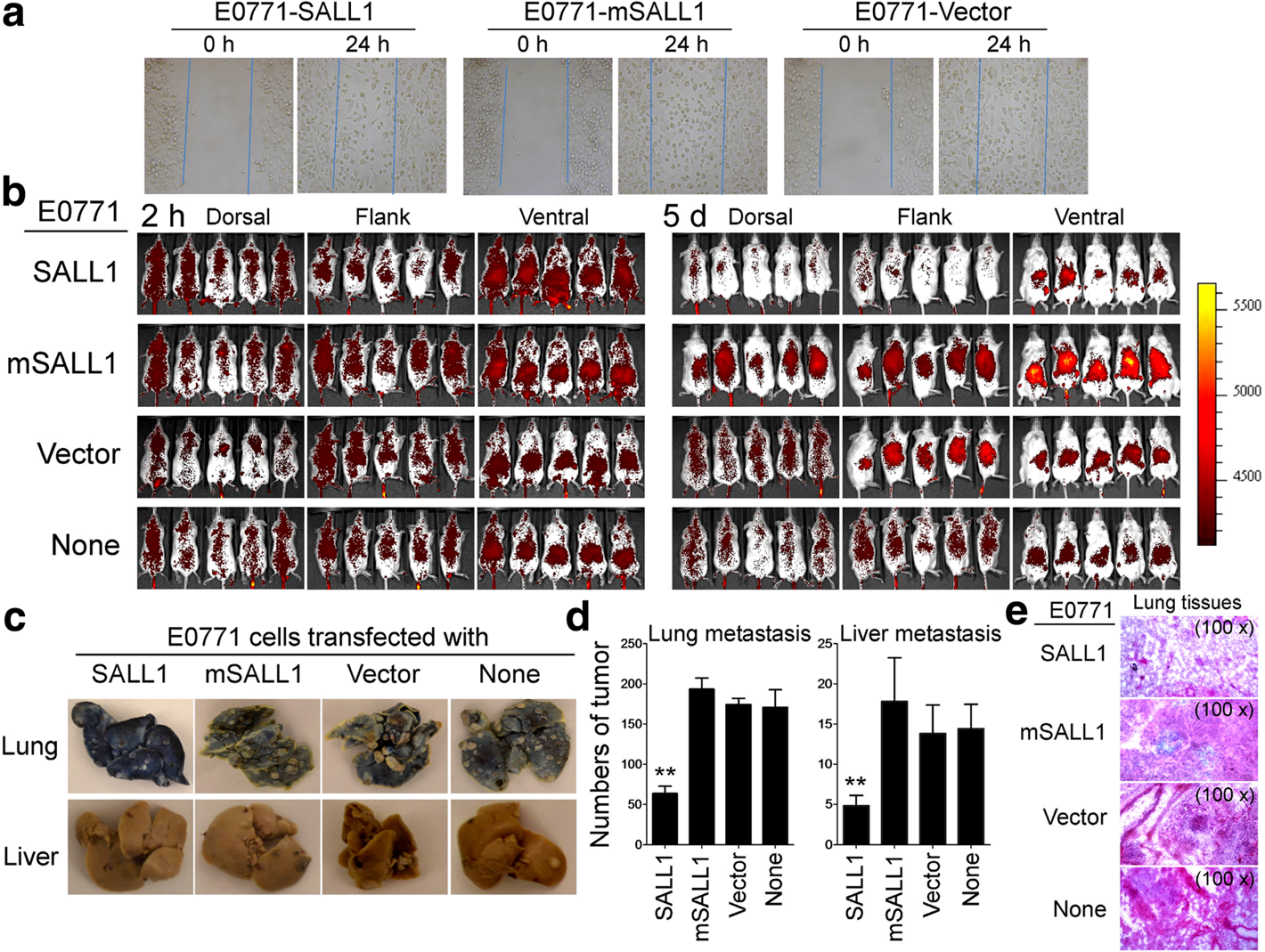
SALL1 over-expression in breast cancer cells inhibited tumor metastasis in vivo. **a** Over-expression of SALL1 in E0771 breast cancer cells significantly inhibited the migration of tumor cells compared with the control mSALL1 and vector-transfected tumor cells in the wound closure assays. Data shown are from three independent experiments with similar results. **b** Over-expression of SALL1 in E0771 breast cancer cells markedly suppressed the tumor cell migration and metastasis in NSG mice. Lentivirus-transfected E0771 tumor cells were stained with VivoTag®680 XL and then injected tail intravenously (5 × 10^4^/mouse) into NSG mice. Mice were imaged with the IVIS Spectrum at different time points following the tumor cell adoptive transfer into NSG mice. Data shown are the dorsal, ventral, and right lateral images of representative of 5 mice per group at 2 h and day 5. Color bar represents signal intensity scales over whole body. **c** and **d** Over-expression of SALL1 in E0771 cells dramatically decreased tumor macro-metastatic/colonized numbers in lung and liver surfaces. Representative images shown in (**c**) are a representative of mouse lungs and livers obtained from the indicated groups at the endpoint of the experiments. Tumor colonized spots were counted and results shown are mean ± SD in (**d**) (*n* = 5 mice per group). **p < 0.01, compared with the control groups transfected with mSALL1 and vector using unpaired t-test. **e** H & E staining on sections from embedded lung tissues showed that high amount of tumor cells infiltrated into lungs obtained from control groups (mSALL1 and vector), but not from the SALL1 transfection group

We next investigated whether over-expression of the SALL1 inhibited breast tumor metastasis. In vitro wound closure assays were utilized to determine the capacity of tumor cell migration after transfection with or without SALL1. We observed that over-expression of SALL1 in E0771 and MCF7 breast tumor cells markedly inhibited the migration of tumor cells compared with the tumor cells alone, or tumor cells transfected with mSALL1 or vector (Fig. 8a and Additional file 1: Figure S7A). We then investigated whether over-expression of SALL1 affected tumor metastasis using previously established adoptive transfer tumor models with a live imaging system [45]. Mouse E0771 tumor cells infected with or without lentivirus carrying SALL1 or control mutated SALL1 gene were labeled with VivoTag®680 XL and then injected intravenously into the tail vein of NSG mice. Tumor cell distribution and metastasis in mice were imaged at dorsal, right lateral and ventral positions with an In Vivo Spectrum Imaging System (IVIS) at different time points post injection. As shown in Fig. 8b and Additional file 1: Figure S7B, at early time points (before 3 days) post tumor cell adoptive transfer, tumor cells randomly migrated into different organs, including spleen, liver and lung with similar signal densities among different groups. The signal density of tumor cells significantly increased and accumulated in these sites in the late time points (after 3 days) post tumor cell transfer among the groups of E0771 tumor cells only, or transfected with mSALL1 or vector. This tumor cell accumulation with strong signal density continued to persist throughout the whole observation period (19 days), indicating accumulated colonization of tumor cells into the lung and liver. However, the signal density from the tumor cells transfected with SALL1 markedly decreased in the late time points (after 3 days) post tumor cell injection, suggesting the lower capacity of tumor metastasis and/or colonization compared with that of the other groups. To further confirm the live imaging results, livers and lungs from the four groups were harvested at day 19 post tumor injection and macro-metastatic tumors were evaluated. As expected, we observed that transferred E0771 tumor cells alone or transfected with mutated SALL1 gene or vector control, grew significantly with metastasis/colonization in lungs and livers (Fig. 8c and d). In contrast, transfection of SALL1 in E0771 cells dramatically decreased tumor macro-metastatic numbers both in lung and in liver surfaces. Furthermore, we confirmed, using histological staining on sections from embedded liver and lung tissues that a high number of tumor cells infiltrated into livers and lungs obtained from control groups of mSALL1 and vector-transfected E0771, but not from the SALL1-transfected tumor group (Fig. 8e and Additional file 1: Figure S7C). Our studies clearly demonstrate that SALL1 is a tumor suppressor in breast cancer and plays a critical role in directing tumorigenesis and metastasis.

## Discussion

SALL2 and SALL4 have been recently recognized as regulators of tumorigenesis [12–15], but little information is known about the role of the SALL1 gene in regulation of tumor biology. In the current study, we showed that SALL1 expression was significantly down-regulated in specific human breast cancer subtypes based on analyses of clinical tumor samples and cell lines. We further demonstrated that SALL1 expression in human and murine breast cancer cells controlled tumor cell growth and proliferation in vitro, and that overexpression of SALL1 inhibited tumorigenesis and metastasis in vivo in breast cancer xenograft models. Importantly, the tumor suppressor function mediated by SALL1 is mechanistically related to cell senescence induction via the recruitment of the NuRD complex in cancer cells. Our studies clearly indicate that SALL1 functions as a tumor suppressor in breast cancer, which could be a novel target for human breast cancer therapy.

A recent paper demonstrated that SALL1 could be a tumor suppressor in human breast cancer, using an in vivo RNAi screen strategy [20]. They further showed that high expression of SALL1 was associated with significantly increased relapse-free survival, overall survival, metastasis-free survival, and tumor-free survival of breast cancer patients. However, whether and how SALL1 regulates human breast cancer is still unclear. Improved understanding of the molecular events should open new avenues for breast cancer clinical therapy. Our group has extensively studied the molecular mechinasms responsible for SALL1-mediated regulation in kidney development, and demonstrated that endogenous SALL1 recruits and binds to the NuRD complex to regulate transcriptional repression and specific developmental processes, such as progenitor cell fate [4, 6, 21–23]. In this study, using both the loss-of-function (either deletion of the conserved NuRD-binding 12-amino acid peptide motif or substitution of the serine with a glutamic acid SALL1), and gain-of-function (mutating the serine to an alanine) strategies in vitro and in vivo studies, we clearly demonstrated that SALL1 also utilizes a similar mechanism as in the developing kidney to recruit the NuRD complex, resulting in the inhibition of tumorigenesis and metastasis in breast cancer [21, 22]. In support of our novel finding, studies from other groups have already shown that the key components of NuRD complex, including MTA1, MTA3, and Mi-2 (CHD4), and other NuRD interacting proteins such as LSD1, directly control the invasive growth, epithelial-to-mesenchymal transition, and metastasis in breast cancer [24–26]. Furthermore, two groups identified frequent somatic mutations in the NuRD component chromodomain helicase DNA-binding protein 4 (CHD4) in an aggressive form of uterine cancer [46, 47]. Importantly, a more recent study demonstrated that loss of CHD4 leads to therapeutic resistance in BRCA2 mutant ovarian cancer [48]. Our current work further suggests a causative link between SALL1 gene regulation, NuRD complex function, and breast cancer pathogenesis. In addition to the recruitment of the NuRD complex, SALL1 may also be involved in the regulation of other oncogenes, such as PTEN and c-Myc [10, 49]. However, we did not observe changes of these two oncogenes in breast cancer cells mediated by SALL1 over-expression (Data not shown). Notably, the MTA1, MTA2 and MTA3 components of the NuRD complex have been shown to play an important role in the ER and HER2 pathways regulating the epithelial-mesenchymal transition (EMT) and tumor cell invasion and metastasis, but they have distinct effects [24, 26]. Given that ER, PR and HER2 expression levels in tumor cells are important prognostic factors for breast cancer outcomes, we also determined whether SALL1 had different expression in breast cancer patients with different ER, PR or HER2 expression statuses. Our results suggested that SALL1 expression in HER2^+^ patients was much higher than that in HER2^−^ patients. In addition, SALL1^+^ cell numbers in ER^−^ patients were significant lower than those in ER^+^ patients. Notably, analyses of TCGA normalized log_2_ transformed breast cancer argilent microarray expression data sets clearly showed a significant decrease of SALL1 gene expression in the basal like breast cancer, as well as in ER^−^, PR^−^ and triple negative breast cancer tissues. To further identify potential mechanism responsible for the down-regulation of SALL1 in breast cancer, we explored promoter methylation status of the SALL1 gene via COSMIC genome browser [27]. Consistent with the previous report showing that the SALL1 promoter was methylated in breast and other epithelial cancers [19], our analysis also demonstrated that the genes were highly methylated in the 2 regions of the SALL1 promoter in the breast cancer tissues. In addition, studies from other groups have demonstrated that SALL1, which is located at 16q12.1 is a region that was shown to undergo loss of heterozygosity (LOH) in breast, prostate, ovarian cancers and in retinoblastoma [50, 51]. We will continue our efforts to identify the molecular interactions and regulatory mechanisms between SALL1, NuRD, and ER, PR and HER2, in the regulation of tumorigenesis and metastasis in breast cancer. In addition, given the multiple functions mediated by different subunits of the NuRD complex, identification of the precise assembly of NuRD components recruited by SALL1 in breast cancer cells will facilitate our understanding the functional role of SALL1 gene in tumor biology.

Besides the recruitment of NuRD complex, our current study also identified senescence induction as a novel mechanism mediated by SALL1 for the regulation of tumor biology and tumorigenesis in breast cancer. Cellular senescence was initially described more than 50 years ago in human fibroblasts with limited passages in cell culture. It is now well known that senescent cells have permanent cell cycle arrest, but remain viable, metabolically active and possess unique transcriptional profiles and gene regulation signatures. There are two major categories of cellular senescence: (1) Replicative senescence (telomere-dependent senescence) occurs due to telomere shortening or dysfunction that triggers a classical DNA-damage response [29, 30]; and (2) Premature senescence (extrinsic senescence or telomere-independent senescence) is induced by a variety of extrinsic forms of stress, such as oxidative stress, DNA damage, and activation of certain oncogenes, as well as some inflammatory cytokines and chemokines [52]. We have recently demonstrated that human Treg cells and tumor cells can also induce ATM-associated DNA damage in responder T cells resulting in T cell senescence [31, 32, 34, 53]. In addition, cellular senescence is now thought to be a tumor suppressive mechanism that could be a harnessed as a possible cancer therapy strategy [52]. In this study, we were the first to show that SALL1 also plays a critical role in control of genome stability, cell-cycle progression and cell fate in breast cancer. Specifically, we observed that SALL1 gene expression in breast cancer strongly suppresses tumor growth and proliferation, as well as induces cell cycle S phase arrest, which is mechanistically independent of apoptosis or cytolysis. We further discovered that SALL1-mediated suppression of breast cancer cells is due to the induction of tumor cell senescence as shown by induction of SA-β-Gal [31, 32, 53]. We identified that ATM-associated DNA damage is responsible for SALL1-mediated breast cancer cell senescence, by analyzing activation of ATM and its related targets, as well as using loss-of-function approaches with a specific pharmacological ATM inhibitor and shRNA. Importantly, we also provide evidence demonstrating that the SALL1-mediated suppression of tumor growth, cell proliferation and induction of tumor cell senescence depends on the endogenous recruitment of NuRD complex in breast cancer cells. The regulation of cell cycle transition and DNA damage responses mediated by the NuRD complex has been well recognized [24, 26, 54]. MTA1 and MTA2 can directly regulate p53 stability and function, leading to growth arrest inhibition and DNA damage response regulation [55]. CHD4 is also as an important regulator of the G1/S cell cycle transition and ATM-associated DNA damage responses [56]. In addition, HDAC1 and HDAC2 regulate the DNA-damage response and cellular senescence [57]. Our previous studies have shown that SALL1 binding with NuRD directly repressed Gbx2, suggesting that Gbx2 is a direct SALL1 target gene [21, 22]. Furthermore, mutating the NuRD binding motif in SALL1 not only prevented binding of NuRD components, but the associated HDAC activity was also completely lost [21, 22]. Gbx2 was shown to be a marker of chemoresistance in triple negative breast cancer [58]. However, how and whether Gbx2 and HDAC involve SALL1-mediated tumor cell DNA damage and senescence is still unknown in the current study. Future studies will continue to focus on the identification of the subunits of NuRD and target genes recruited by SALL1 in breast cancer cells responsible for the regulation of DNA-damage response and senescence induction. Interestingly, one study suggested that SALL2 directly binds to the p21 promoter promoting cell cycle arrest and inhibiting cell growth [13]. SALL1 binds the p21 promoter and represses luciferase activity driven by this promoter in a NuRD dependent manner (Our unpublished observations). Consistent with this finding, it has been shown that CHD4 also binds the p21 promoter and inhibits expression of this cell cycle gene [59]. Therefore, the ability of SALL1 to directly modulate cell cycle regulatory molecules, such as p21, is another potential mechanism that needs to be explored.

Dissection of the unique molecular signaling responsible for SALL1-mediated tumor suppression is another challenge. Our studies clearly showed that SALL1 expression in breast tumor cells selectively modulated the MAPK p38 and ERK1/2, as well as mTOR signaling pathways in tumor cells. In addition, the loss-of-function studies with specific pharmacological inhibitors and lentivirus-based shRNAs further indicated that SALL1-mediaed tumor suppression and senescence induction is controlled by both MAPK and mTOR signaling pathways. It is well recognized that MAPK signaling pathways play a major role in regulating cell cycle re-entry, oncogenic ras-induced senescence and G1 cell cycle arrest [35, 36]. Our recent studies further demonstrated that MAPK ERK1/2 and p38 signaling controls the molecular process of human CD4^+^CD25^hi^FoxP3^+^ Treg-induced responder T cell senescence [32]. In addition to MAPK signaling, mTOR kinase signaling activation is important for tumor cell proliferation and senescence induction [37]. mTOR signaling is also involved in the oncogene-induced DNA damage responses and cell senescence [39–41]. Our current studies further identified important roles of these two signaling pathways in SALL1-mediated regulation in breast cancer cells. However, the results presented here are different from our previous observations showing that SALL1 induces Wnt signaling in the developing kidney [4]. Interestingly, we did not find activation of Wnt signaling in breast cancer MCF-7 and E0771 cells induced by SALL1 over-expression. These results suggest that the molecular signaling utilized by SALL1 promoting its tumor suppressor function is different from that in the regulation of organ development. Further dissection of how MAPK signaling and mTOR signaling cooperate and identification of unique adaptor molecules controlling SALL1 biological functions in tumor cells will be critical preludes for the application of SALL1 and tumor senescence as new targets for tumor therapeutic interventions.

## Conclusion

We identified SALL1 as a novel tumor suppressor in breast cancer. We demonstrated that SALL1 can induce tumor cell senescence as a novel mechanism of tumor suppressor function. This molecular process acts through NuRD recruitment and is controlled by the MAPK and mTOR signaling pathways. These studies not only reveal a novel role of SALL1 in breast cancer biology, but also provide the mechanistic and causative links among SALL1 regulation, cellular senescence, NuRD, as well as MAPK and mTOR signaling pathways. These important aspects should provide new insights relevant for the development of novel therapeutic strategies in human breast cancer and other cancers as well.

## Methods

### Human samples and cell lines

Tumor samples were obtained from breast cancer patients treated at the Department of Surgery, Saint Louis University from 2004 to 2015 who have given informed consents for enrollment in a prospective tumor procurement protocol approved by the Saint Louis University Institutional Review Board. Paired fresh tumor tissues and normal breast tissues were obtained perioperatively and snap frozen in liquid nitrogen. In addition, fresh-frozen metastatic cutaneous melanoma tumor tissues were also collected as controls for this study. Breast tumor cell lines (human MDA-MB-231, MCF7, BC80, 31, 30, 29, 16, 12, 10, and murine 4 T1 and E0771), Melanoma cell lines (Mel1938, Mel1586, Mel1860, Mel1363, Mel1526 and Mel1628, and murine B16F0), prostate cell line PC3 and DU145, colon cancer cell line SW480 and lymphoma L428 and L504, as well as normal breast cells and fibroblast cells, were either obtained from the American Tissue Culture Collection (ATCC) or established by our group, and maintained in RPMI 1640 medium containing 10% fetal calf serum (FCS) and penicillin-streptomycin (Invitrogen, Inc. San Diego, CA).

### Plasmid constructs

Full length flu-tagged SALL1 wild type and mutant constructs cloned into pcDNA3.1 were prepared as previously described [21]. Point mutants were created by PCR-mediated site directed mutagenesis using QuikChange (Stratagene). The amplified PCR products were cloned into lentivirus vector pCDH-CMV-EF1-GFP. The nucleotide sequences of all constructs were verified by DNA sequencing.

### Immunohistochemical staining of SALL1 and quantification method

The cell populations of SALL1^+^ cells in cancer and normal tissues (frozen sections) were determined using immunohistochemical staining with the Histostain®-Plus 3rd Gen IHC Detection Kit (Invitrogen, CA), as we described previously [31]. Immunohistochemical reactions were performed using either mouse monoclonal or rabbit polyclonal antibodies against SALL1 at dilution of 1:1500. Controls were performed by incubating slides with the isotype control antibody instead of primary antibodies, or second antibody alone. SALL1^+^ cells in tissues were evaluated manually using a computerized image system composed of a Leica ICC50 camera system equipped on a Leica DM750 microscope (North Central Instruments, Minneapolis, MN). Photographs were obtained from 20 randomly selected areas within the tumor tissues of 10 cancer nest areas and 10 cancer stroma areas at a high-power magnification (400 ×). Both cancer nest and stroma areas were counted and summed, and the means of positive cell numbers per field reported.

### Reverse-transcription PCR analysis

Total RNA was extracted from tumor or normal tissues and cell lines using Trizol reagent (Invitrogen), and cDNA was transcribed using a SuperScript II RT kit (Invitrogen), both according to manufacturers’ instructions. mRNA expressions of each gene were determined by reverse-transcription PCR using specific primers, and mRNA levels in each samples were normalized to the relative quantity of Glyceraldehyde-3-phosphate dehydrogenase (GAPDH). All samples were run in triplicate. The primers for each gene used were as following:

*SALL1*: 5’ TGATGTAGCCAGCATGT 3′ and 5’ AAAGAATTCAGCGCAGCAC 3’.

*SALL2*: 5’ CCAAGAGTAAAGCGGATGAGA 3′ and 5’ AGTAAGCAGTGCCCAACTCG 3’.

*SALL3*: 5’ TGGGCCTTCGCTTACTAAAG 3′ and ACAGCAGTGGCAGCTGAAG 3’.

*SALL4*: 5’ AGCAGCCTCAGCAGCTACC 3′ and 5’ AAGAACTCGGCACAGCATTT 3’.

*Cyclin A2*: 5’ GGATGGTAGTTTTGAGTCACCAC 3′ and 5’ CACGAGGATAGCTCTCATACTGT 3’.

*Cyclin B1*: 5’ AACTTTCGCCTGAGCCTATTTT 3′ and 5’ TTGGTCTGACTGCTTGCTCTT 3’.

*Cyclin D1*: 5’ CAATGACCCCGCACGATTTC 3′ and 5’ CATGGAGGGCGGATTGGAA 3’.

*Cyclin E1*: 5’ ACCGGTATATGGCGACACAAGAA 3′ and 5’ TCACATACGCAAACTGGTGCAA 3’.

*CDK2*: 5’ GCATCTTTGCTGAGATGGTGACTC 3′ and 5’ AGTAACTCCTGGCCACACCA 3’.

*CDK4*: 5’ CATTCTGGTGACAAGTGGTGG 3′ and 5’ TCGGCTTCAGAGTTTCCACAG 3’.

*CDK6*: 5’ CCAGATGGCTCTAACCTCAGT 3′ and 5 ‘AACTTCCACGAAAAAGAGGCTT 3′.

### Cell growth and functional proliferation assay

Tumor cell lines were plated at 2 × 10^4^/well in 24 wells and transfected with one of the following plasmids: pcDNA3.1-SALL1, pcDNA3.1-SALL4, and pcDNA3.1. Cell growth was evaluated at different time points by counting cell numbers. Proliferation assays were performed as previously described [60]. In brief, different numbers of tumor cells (2 × 10^4^, 5 × 10^4^, or 1 × 10^5^) transfected with or without the related genes were cultured in 96-well plates in cell assay medium containing 2% FCS. After 56 h of culture, [^3^H]-thymidine was added at a final concentration of 1 μCi/well, followed by an additional 16 h of culture. The incorporation of [^3^H]-thymidine was measured with a liquid scintillation counter.

### Cell cycle and apoptosis assays

Transfected cells were cultured for 72 h and apoptosis was analyzed after staining with PE-labeled Annexin V and 7-AAD (BD Biosciences, San Diego, CA). For cell cycle analysis, transfected cells were fixed with 70% ethanol overnight, washed with PBS and incubated with propidium iodide (10 μg/ml) and RNase A (100 μg/ml). Untransfected cells served as controls. All the stained cells were analyzed on a FACSCalibur (BD Bioscience) and the data were analyzed with FlowJo software (Tree Star, Ashland, OR).

### Colony formation assay

Five thousand per well of tumor cells infected with lentivirus carrying shRNA against SALL1 or control scramble shRNA, or transfected with SALL1, were seeded in 6-well plates with 0.4% agar for culture. Cell colonies were stained with crystal violet and counted after 2–3 weeks of culture.

### Senescence associated β-galactosidase (SA-β-gal) staining

Senescence associated β-Galactosidase (SA-β-Gal) activity in tumor cells was detected as we previously described [31, 32]. Briefly, tumor cell lines were transfected with or without plasmids and cultured for 3 or 5 days. Cells were washed in PBS (pH 7.2), fixed in 3% formaldehyde, and followed to incubate overnight at 37 °C with freshly prepared SA-β-Gal staining solution (1 mg/ml X-gal, 5 mM K_3_Fe[CN]_6_, 5 mM K_4_Fe[CN]_6_, 2 mM MgCl_2_ in PBS at pH 6.0). The stained cells were washed with H_2_O and examined with a microscope. For some experiments, SA-β-Gal^+^ populations were determined in the transfected tumor cells after exposure to various inhibitors or combined transfection with shRNAs: ATM inhibitor KU55933 (20 μM, Tocris Bioscience); mTOR inhibitor Rapmycin (5 μM, Sigma); MAPK inhibitors U0126 (10 μM), SB203580 (10 μM) and SP600125 (10 μM), or PI3 Kinase inhibitor Wortminnin (10 μM) (Calbiochemistry), or transfection with shRNAs against p38, ERK and mTOR, for 3 or 5 days. The treated tumor cells were then detected for SA-β-Gal expression.

### Western-blotting analysis and protein interaction assays

Breast cancer cells transfected with or without plasmids pcDNA3.1-SALL1 or pcDNA3.1-mSALL1, were cultured for 0, 24 h, 48 h and 72 h. Whole cell lysates were prepared for western blotting. The antibodies used in western blotting are as follows: anti-SALL1, anti-ERK, anti-phospho-ERK, anti-p38, anti-phospho-p38, anti-JNK, anti-phospho-JNK, anti-phospho-p53 (ser15), anti-mTOR, anti-phospho-mTOR; anti-P70S6K, anti-phospho-P70S6K; anti-4E-BP1, anti-phospho-4E-BP1, anti-PTEN, anti-phospho-PTEN and anti-GAPDH rabbit polyclonal antibodies (Cell Signaling Technology, Danvers, MA).

Protein interaction analysis of NuRD complex members with SALL1 (2–137) was performed as previously described [21, 22]*. In brief,* MCF-7 breast tumor cells were transfected with plasmids pEBG-SALL1, pEBG-SALL1-S2A, and pEBG-SALL1-S2E, and allowed to expression GST-SALL1 fusion proteins for 48–72 h. Cells were incubated for 1 h on ice in lysis buffer (1% Triton X-100, 200 mM sucrose, 50 mM Tris pH 7.4, plus protease cokatail) and the cell suspension was disrupted by sonication. GST-SALL1 fusions and associated protein complexes were isolated by precipitation of 50 μg of total protein with glutathione-Sepahrose beads (Amersham Biosciences) for 2 h at 4 °C. Protein pulldowns were separated by SDS-PAGE and proteins detected by western blot. Primary antibodies were all used at 1:1000 dilution and included: rabbit anti-SALL1, rabbit anti-Mta2 (Abcam, ab 8106), mouse anti-RbAp48 (GeneTex, GTX 70237), rabbit anti-Hdac1 (Abcam, ab 19,845), rabbit anti-Mbd3 (Abcam, ab 157,464). Secondary antibodies were used at 1:10,000 included: goat anti-rabbit (Sigma, A0545) and rabbit anti-mouse (Jackson immune Research 315–035-048).

### Flow cytometry analysis

The expression of DNA damage response markers on tumor cells were determined by FACS analysis after staining with anti-human specific antibodies conjugated with either PE or FITC. These human antibodies included: anti-phosphorylated H2Ax, anti-phosphorylated p53bp, and anti-phosphorylated ATM, which were purchased from Cell Signaling Technology or BD Biosciences. All stained cells were analyzed on a FACSCalibur flow cytometer (BD Bioscience) and data analyzed with FlowJo software (Tree Star).

### Cell migration and wound healing assay

Breast cancer E0771 and MCF-7 tumor cells transfected with Lenti-*SALL1*, Lenti-m*SALL*1 or vector, were plated in 6-well plates and grown to confluence. A wound area was generated by scraping cells with a 200 μl pipette tip across the entire diameter of the dish and extensively rinsed with the medium to remove all cellular debris. Low-serum RPMI 1640 with mitomycin (2 μg/ml) was then added to inhibit cell proliferation during the experiment and the closing of the wound was observed at different time points.

### Lentivirus-shRNA generation and gene knockdown in tumor cells

The methods for design and construction of shRNA specific for ERK1, ERK2, P38α, JNK1 and mTOR or scrambled lenti-shRNAs, and generation of recombinant lentivirus carrying GFP and shRNA, have been described previously [60]. shRNAs specific for ATM (TRCN0000194861, TRCN0000039951 and TRCN0000360327), mouse SALL1 (TRCN0000238153, TRCN0000238154 and TRCN0000238155) and human SALL1 (TRCN0000003956, TRCN0000003957 and TRCN0000003958), were purchased from Sigma Aldrich. For lentivirus infection, concentrated lentiviral supernatant with a multiplicity of infection (MOI) of 5–10 in a total volume of 0.5 ml culture medium was added to the tumor cells growing in 24 well plates containing 8 μg/ml polybrene (Sigma), and then centrifuged at 1000 x g for 1 h at room temperature. The infected tumor cells were then transfected with or without pcDNA3.1-*SALL-1*, and induction of senescence was determined.

### In vivo tumorigenesis and metastasis studies

NOD-scid IL2Rγ^null^ (NSG, 6–8 weeks) immunodeficient mice were purchased from The Jackson Laboratory and maintained in the institutional animal facility. All animal studies have been approved by the Institutional Animal Care Committee. For tumorigenesis studies, mouse E0771 (2 × 10^5^/mouse) and B16F0 (1 × 10^5^/mouse) tumor cells infected with lentivirus carrying SALL1, mSALL-1 or vector, were subcutaneously injected into NSG mice. Five mice were included in each group. Tumor size was measured with calipers every 2–3 days. Tumor volume was calculated on the basis of two-dimensional measurements. At the end of experiments, the mice were sacrificed and tumors were isolated and weighted. Furthermore, tumor tissues were embedded into OCT and prepared for cryostat sections (4~ 8 μm), and SA-β-Gal expression was assayed, as described above.

For tumor metastasis studies, lentivirus-transfected E0771 tumor cells were incubated with 100 μg/ml of VivoTag®680 XL (PerkinElmer) for 30 min. Stained tumor cells were washed and then injected intravenously into the tail vein (5 × 10^4^/mouse in 200 μl of buffered saline) into NSG mice. Five mice were included in each group. Mice were imaged with an In Vivo Spectrum Imaging System (IVIS) (Caliper Life Science) at 120 min, and 1, 3, 5, 7, 10, 14, 17 and 19 days post injection. The appropriate filter set for VivoTag®680 XL imaging of 665 nm excitation and 688 nm emission was used. Mice were imaged in the dorsal, right lateral and ventral positions at all the time points. Livers and lungs were harvested at 19 days post injection and stained with 15% black India ink. Visible lung and liver surface macro-metastatic appeared as white spots and were counted using a dissecting microscope. Lungs were collected and fixed in 10% formalin. For tissue morphology and metastasis evaluation, liver and lung tissues were embedded into OCT and frozen sections (4~ 8 μm) were prepared and stained with hematoxylin and eosin (H & E).

### Statistical analysis

Statistical analysis was performed with GraphPad Prism5 software. Unless indicated otherwise, data are expressed as mean ± standard deviation (SD). For public database analysis, The Cancer Genome Atlas (TCGA) normalized log_2_ transformed breast cancer Argilent microarray expression data sets and methylation database were downloaded from the cBioPortal (http://www.cbioportal.org/) [27] and used to compare SALL1 mRNA expression among the different breast cancer subtypes and solid normal tissues, as well as analyze methylation difference of SALL1 promoter in COSMIC between the primary tumor in breast cancer and solid normal tissues. The Mann-Whitney U test was utilized for statistical analysis of association between SALL1 expression and breast cancer subtypes. P valve < 0.05 was considered statistically significant. For multiple group comparison in vivo studies, the one-way analysis of variance (ANOVA) was used, followed by the Dunnett’s test for comparing experimental groups against a single control. For single comparison between two groups, paired Student’s *t* test was used. Nonparametric *t*-test was chosen if the sample size was too small and did not fit a Gaussian distribution.

## Additional file

Additional file 1: **Figure S1.** SALL1 and SALL4 expression levels in different types of human cancers. **(A)** SALL1 expression in tumor cells in breast cancer and melanoma tissues was determined using the immunohistochemical staining. Numbers of SALL1^+^ tumor cells in melanoma tissues were much higher than those in breast cancer tissues. Expression level of each dot shown in the right panel is the average numbers of SALL1^+^ cells per high field (400 x) in each tissue sample. The median number of SALL1^+^ cells in each group is shown as a horizontal line. Significance was determined by unpaired T test. **(B)** The COSMIC analysis shows the mutations and the promoter methylation status of SALL1 gene in breast cancer tissues. **(C)** SALL1 promoter genes are highly methylated in the primary breast cancer tissues comparison with solid normal tissues. The 2 specific molecular probes which span the proximal promoter region of SALL1 gene were selected based on the information in COSMIC. The box plots showed that the methylation β value of particular promoter regions in the tissues. N indicated the number of sample size. **P < 0.01 between 2 groups with the Mann-Whitney U test. **(D)** and (E) Gene expression levels of SALL4 in different cancer cell lines (in D) and in tumor tissues (in E) using Real-time PCR analyses. Tumor cell lines include breast cancer (human MDA, MCF7, BC80, 31, 30, 29, 16, and murine 4 T1 and E0771), melanoma (human Mel1938, Mel1586, Mel1860, Mel1363, Mel1526 and Mel1628, and murine B16F0), prostate cancer (PC3 and DU145), colon cancer (SW480), and lymphoma (L428 and L504). Normal breast cell lines (BN6 and BN16), fibroblasts (F163, F160, F158 and F112), 293 T cells, and normal breast tissues were included as controls. mRNA levels in each cancer cell line and tumor tissue were normalized to the relative quantity of GAPDH expression and then adjusted to the express levels in 293 T cells (set as 1). Results shown in the histogram are mean ± SD from three independent experiments. **Figure S2.** The inhibition of breast cancer cell proliferation and growth mediated by SALL1 expression is not due to the induction of apoptosis. **(A)** and **(B)** Transfection of SALL1 but not SALL4 significantly inhibited prostate cancer PC3 cell growth and proliferation. PC3 tumor cells transfected with or without plasmids pcDNA3.1-SALL1, pcDNA3.1-SALL4, and pcDNA3.1, were cultured at a starting number of 2 × 10^4^/well in 24 wells (A), or 1 × 10^4^/well in 96-well plates (B). Cell growth was evaluated at different time points by counting cell number (in A), and cell proliferation was determined using [3H]-thymidine assays (in B). Data are mean ± SD from three independent experiments with similar results. **p < 0.01 compared with the vector control group. **(C)** Transfected breast cancer and melanoma cells were cultured for additional 24 h. Apoptosis in transfected tumor cells was analyzed after staining with PE-labeled Annexin V and 7-AAD. Data shown are representative of three independent experiments with similar results. **Figure S3.** Real-time PCR analyses of mRNA expression levels of the cell cycle regulatory genes in breast cancer MDA-MB-231 cells after transfection with SALL1. MDA-MB-231 tumor cells were transfected with SALL1 for 72 h and then RT-PCR for gene expressions was performed. The expression level of each gene was normalized to GAPDH expression and adjusted to the levels in vector-transfected tumor cells. Data show mean ± SD from three independent experiments with similar results. **Figure S4.** SALL1 over-expression in breast cancer cells induces tumor cell senescence. **(A)** Transfection of SALL1, but not SALL4 in MCF-7 and E0771 breast cancer cells significantly induced the increased SA-β-Gal^+^ cell populations. In contrast, over-expression of SALL1 in B16F0 cells did not induce senescent cells. Transfected tumor cells were cultured for additional 3 days, and senescent cells were analyzed using the SA-β-Gal activity assay. Data shown are mean ± SD from three independent experiments with similar results. **p < 0.01 compared with the vector group. **(B)** SALL1 expression in breast cancer cells induced expression of phosphorylated active of ATM in the transfected cells. Transfected tumor cells were analyzed for the expression of p-ATM expression after culture for 24 h using the FACS analysis. **Figure S5.** SALL1 proteins expressed in transfected breast cancer cells. Wild type SALL1 is shown at the top. Red represents the 12 amino acid region that mediates NuRD complex recruitment. Blue ovals are C2H2 zinc fingers. In mutant mSALL1, the 12 amino acid NuRD recruitment domain is deleted. In SALL1-S2E, the serine at position 2 is mutated to a glutamic acid. This phosphomimetic mutation disrupts NuRD recruitment, similar to the deletion mutant. In SALL1-S2A, the serine at position 2 is mutated to an alanine. This substitution prevents the inactivating serine phosphorylation, thereby enhancing SALL1 mediated NuRD recruitment and its effects on gene transcription. **Figure S6.** Real-time PCR analyses of mRNA expression levels of the other SALL family members in breast cancer cells after transfection with SALL1. MCF7 and E0771 tumor cells were transfected with SALL1 for 24 h and then RT-PCR was performed. The expression level of each gene was normalized to GAPDH expression and adjusted to the levels in vector-transfected tumor cells. Data show mean ± SD from three independent experiments with similar results. **Figure S7.** SALL1 over-expression in breast cancer cells inhibited tumor metastasis in vivo. **(A)** Over-expression of SALL1 in MCF-7 breast cancer cells significantly inhibited the migration of tumor cells compared with the control mSALL1 and vector-transfected tumor cells in the wound closure assays. Data shown are from three independent experiments with similar results. **(B)** Over-expression of SALL1 in E0771 breast cancer cells markedly suppressed the tumor cell migration and metastasis in NSG mice. Lentivirus-transfected E0771 tumor cells were stained with VivoTag®680 XL and then injected tail intravenously (5 × 10^4^/mouse) into NSG mice. Mice were imaged with the IVIS Spectrum at different time points following the tumor cell adoptive transfer. Data shown are the dorsal, ventral, and right lateral images of representative of 5 mice per group at day 3 and day 10. **(C)** H & E staining on sections from embedded liver tissues showed that high amount of tumor cells infiltrated into livers obtained from control groups (mSALL1 and vector), but not from the SALL1 transfection group. (PDF 11758 kb)
