# Correction to: SALL1 functions as a tumor suppressor in breast cancer by regulating cancer cell senescence and metastasis through the NuRD complex

**DOI:** 10.1186/s12943-022-01554-4

**Published:** 2022-03-31

**Authors:** Chunling Ma, Fang Wang, Bing Han, Xiaoli Zhong, Fusheng Si, Jian Ye, Eddy C. Hsueh, Lynn Robbins, Susan M. Kiefer, Yanping Zhang, Pamela Hunborg, Mark A. Varvares, Michael Rauchman, Guangyong Peng

**Affiliations:** 1 Department of Internal Medicine, Saint Louis University School of Medicine, Saint Louis, MO 63104 USA; 2 Department of Laboratory Medicine, Women & Children’s Hospital of Linyi, Shandong Medical College, Linyi, 276000 People’s Republic of China; 3 Department of Laboratory Medicine, The First Affiliated Hospital of Nanjing Medical University, Nanjing, 210029 People’s Republic of China; 4 Department of Obstetrics and Gynecology, Qilu Hospital of Shandong University, Jinan, 250012 People’s Republic of China; 5 Department of Surgery, Saint Louis University School of Medicine, Saint Louis, MO 63104 USA; 6 VA Saint Louis Health Care System, John Cochran Division, St. Louis, MO 63106 USA; 7 Department of Medicine, Washington University, Saint. Louis, MO 63110 USA; 8 Department of Otolaryngology, Saint Louis University School of Medicine, Saint Louis, MO 63110 USA; 9 Department of Otolaryngology, Harvard Medical School, Boston, MA 02114 USA

**Correction to: Mol Cancer 17, 78 (2018)**

**https://doi.org/10.1186/s12943-018-0824-y**

Following publication of the original article [1], an error was identified in the images presented in Fig. 4; specifically: Fig. 4a: incorrect image was used for E0771 cells transfected with SALL1.

**Fig. 4:**
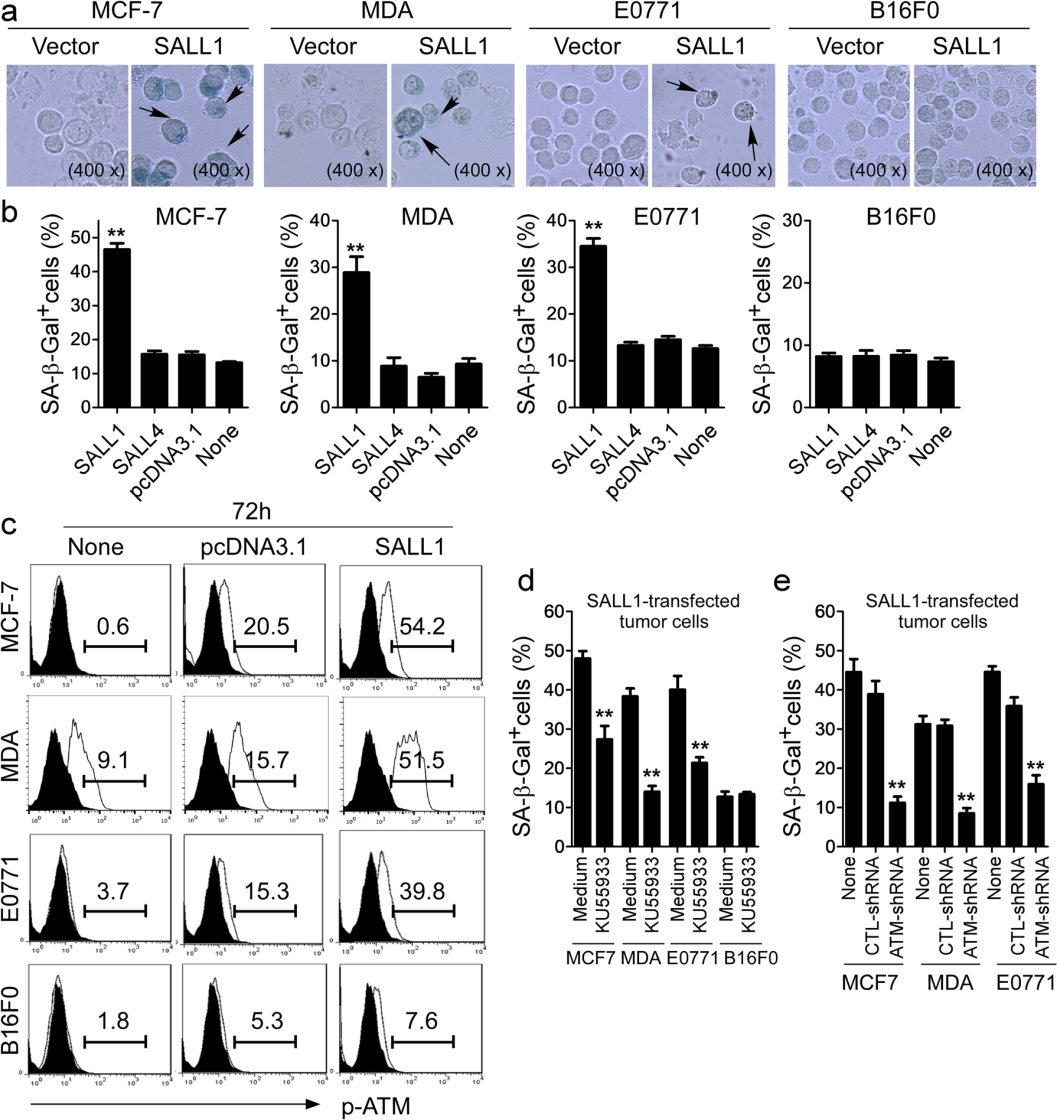
SALL1 over-expression in breast cancer cells induces tumor cell senescence and ATM-associated DNA damage response. **a** and **b** Transfection of SALL1, but not SALL4 in MCF-7, MDA-MB-231 and E0771 breast cancer cells significantly induced the increased SA-β-Gal^+^ cells. In contrast, over-expression of SALL1 in B16F0 melanoma cells did not increase senescent cell populations. Transfected tumor cells were cultured for an additional 5 days. Senescent cells were analyzed using the SA-β-Gal activity assay and the SA-β-Gal^+^ tumor cells were identified with dark blue granules as indicated by the arrows (in **a**). Data shown in (**b**) are mean ± SD from three independent experiments with similar results. **p < 0.01 compared with the vector control group. **c** SALL1 expression in breast cancer cells induced phosphorylated activation of ATM in the transfected cells. Transfected tumor cells were determined for the p-ATM expression after culture for 3 additional days using FACS analyses. **d** Pretreatment of breast cancer cells with an ATM specific inhibitor KU55933 significantly prevented the induction of tumor cell senescence induced by SALL1 expression. Tumor cells were pretreated with or without KU55933 (20 μM) for 1 day, and then transfected with SALL1. SA-β-Gal expression in the transfected tumor cells was determined with SA-β-Gal staining after culture for 3 additional days. Data shown are mean ± SD from three independent experiments, and paired t-test was performed. **p < 0.01, compared with the medium only control group. **e** Knockdown of ATM gene by shRNAs in MCF-7, MDA-MB-231 and E0771 cells dramatically blocked SALL1-induced tumor cell senescence. Breast cancer cells were transfected with lenti-shRNAs specific for ATM or control shRNAs. Transduced cancer cells were then transfected with SALL1 and cultured for 5 days. The SA-β-Gal^+^ cancer cells were determined. **p < 0.01, compared with the group transduced with the control shRNA. Data shown are representative of three independent experiments with similar results

The corrected figure is given here. The correction does not have any effect on the results or conclusions of the paper.
