# Supplementary material for: SALL1 functions as a tumor suppressor in breast cancer by regulating cancer cell senescence and metastasis through the NuRD complex
Source: Mol Cancer. 2018 Apr 6;17:78. doi: 10.1186/s12943-018-0824-y (PMC5889587; doi:10.1186/s12943-018-0824-y)

Figure 1. Ma et al.

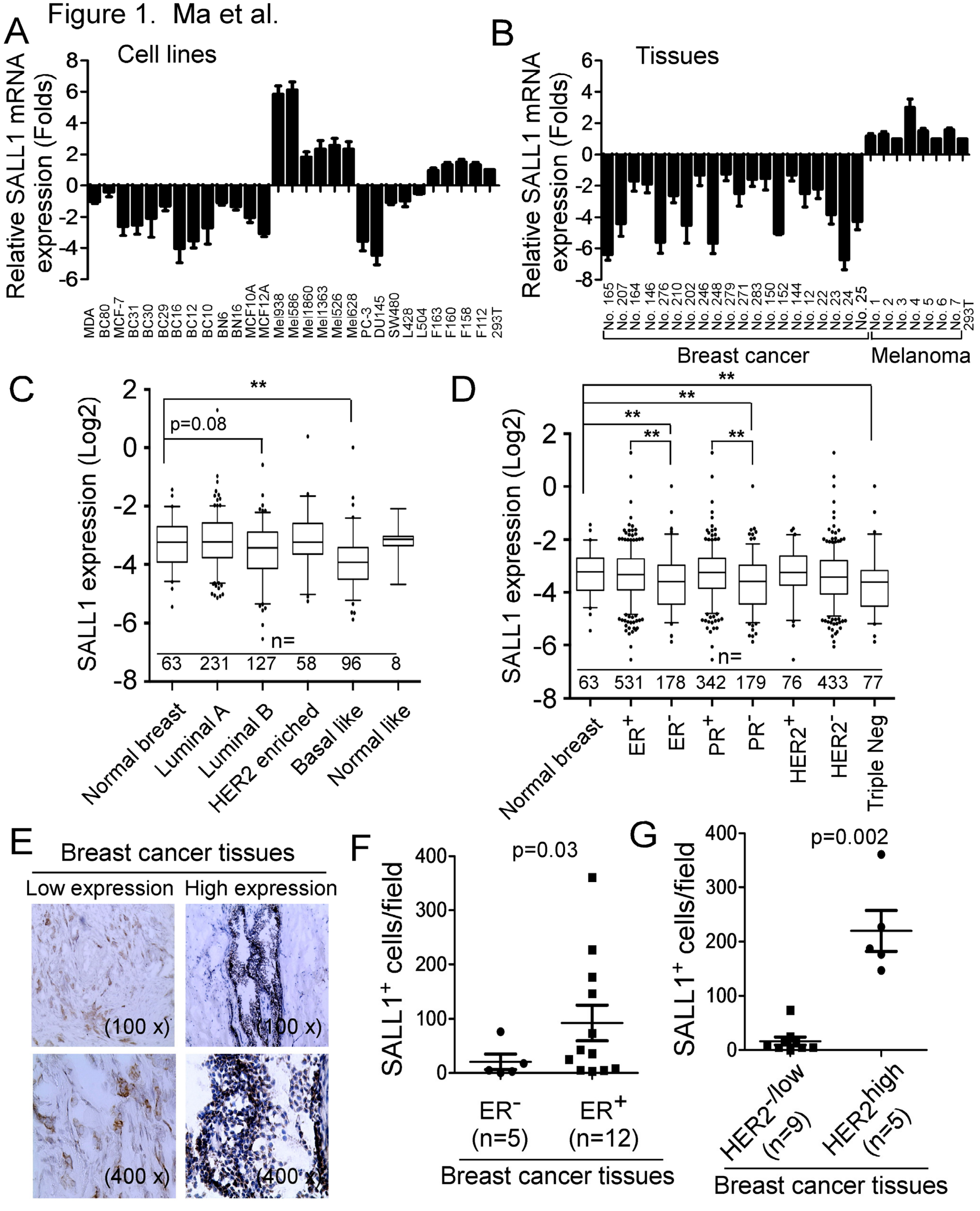

Figure 2. Ma et al.

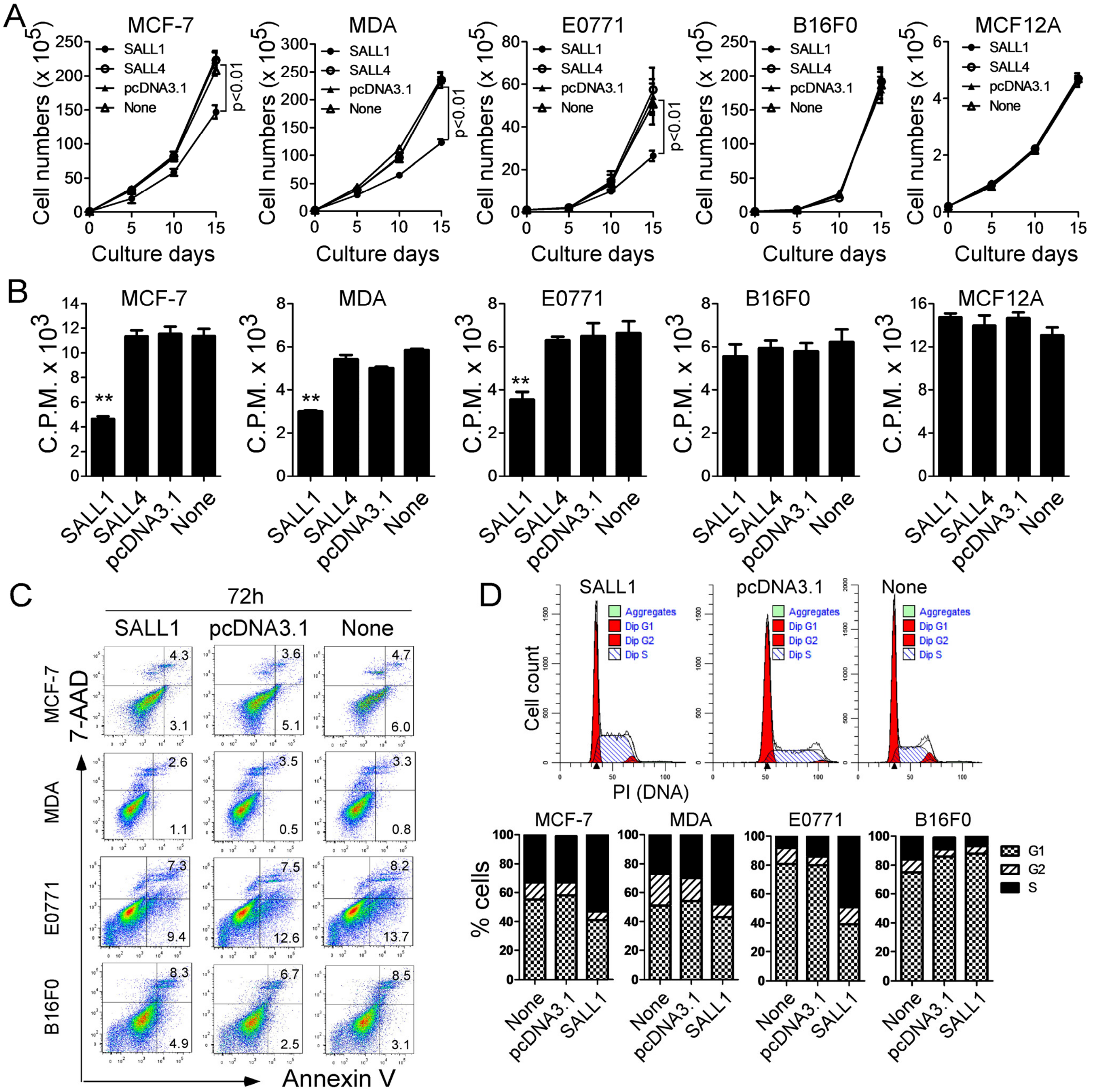

Figure 3. Ma et al.

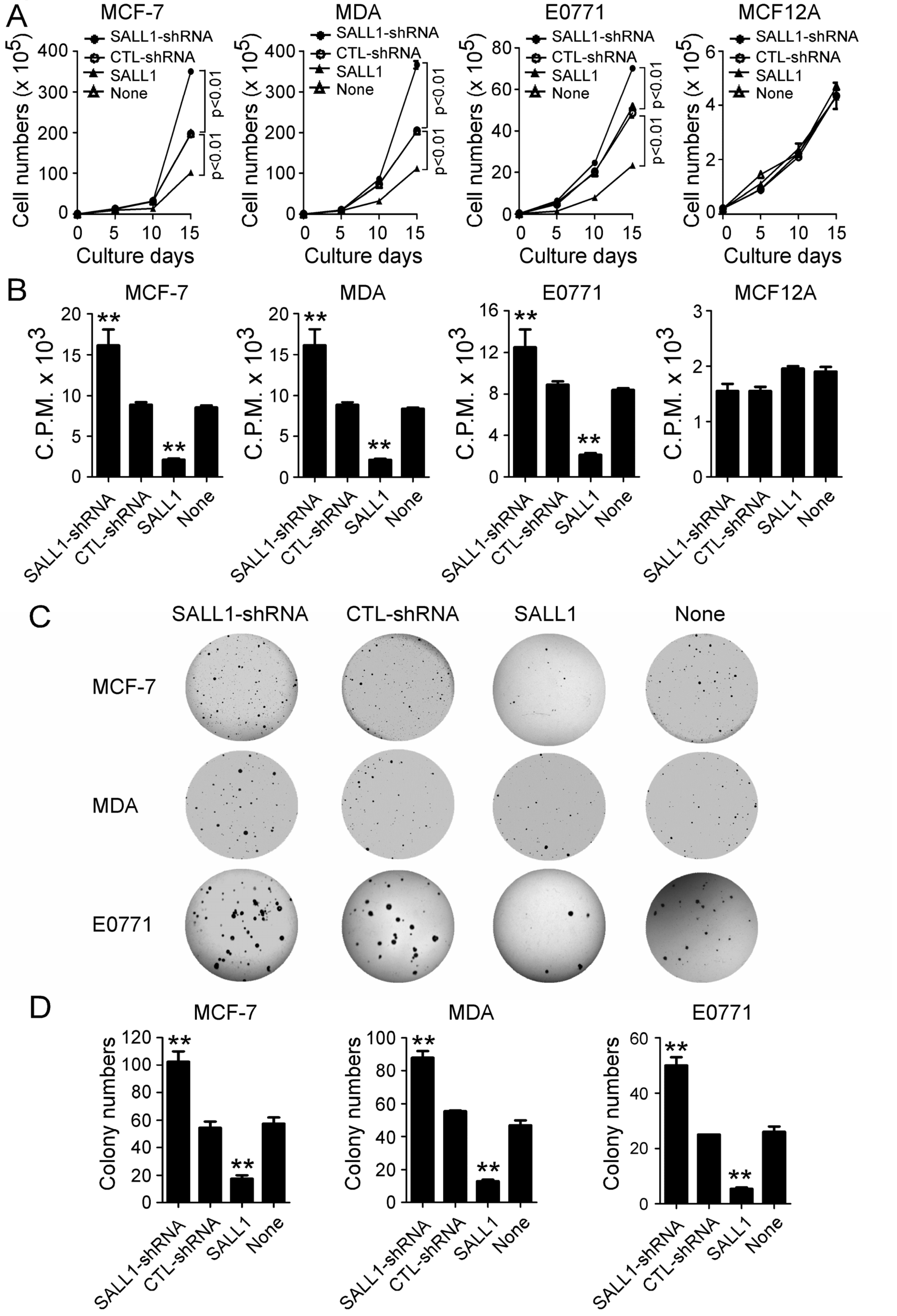

Figure 4. Ma et al.

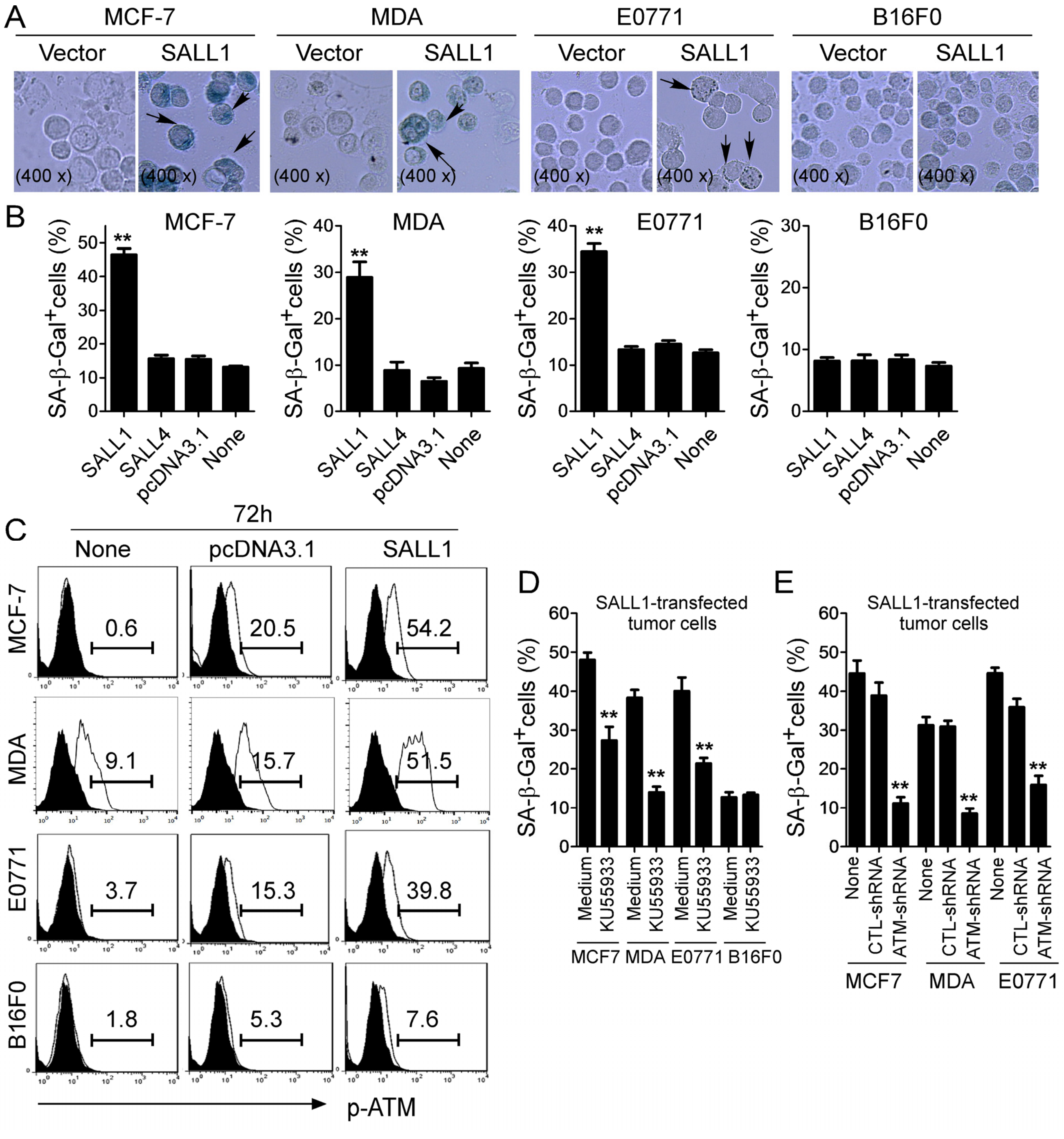

Figure 5. Ma et al.

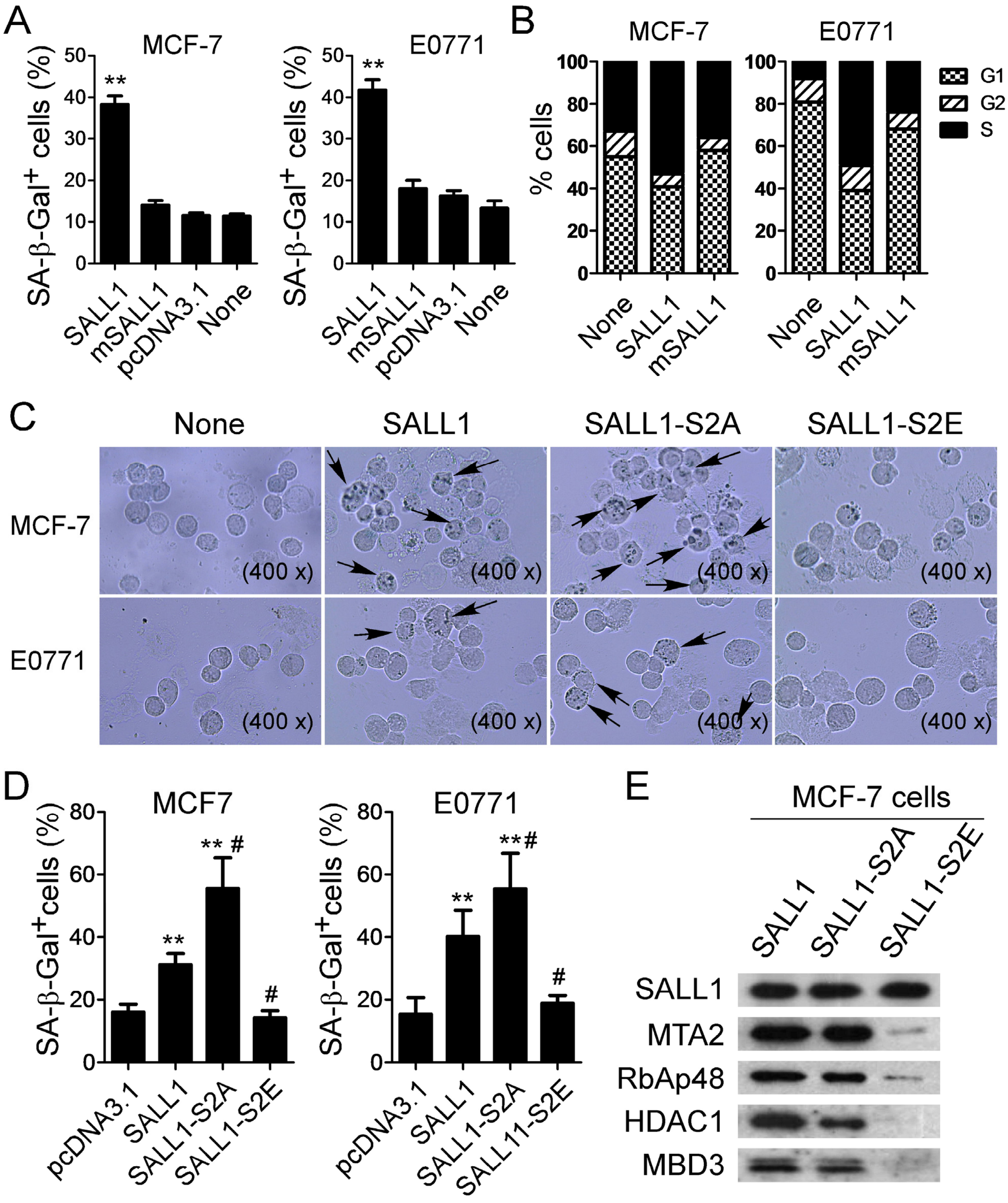

Figure 6. Ma et al.

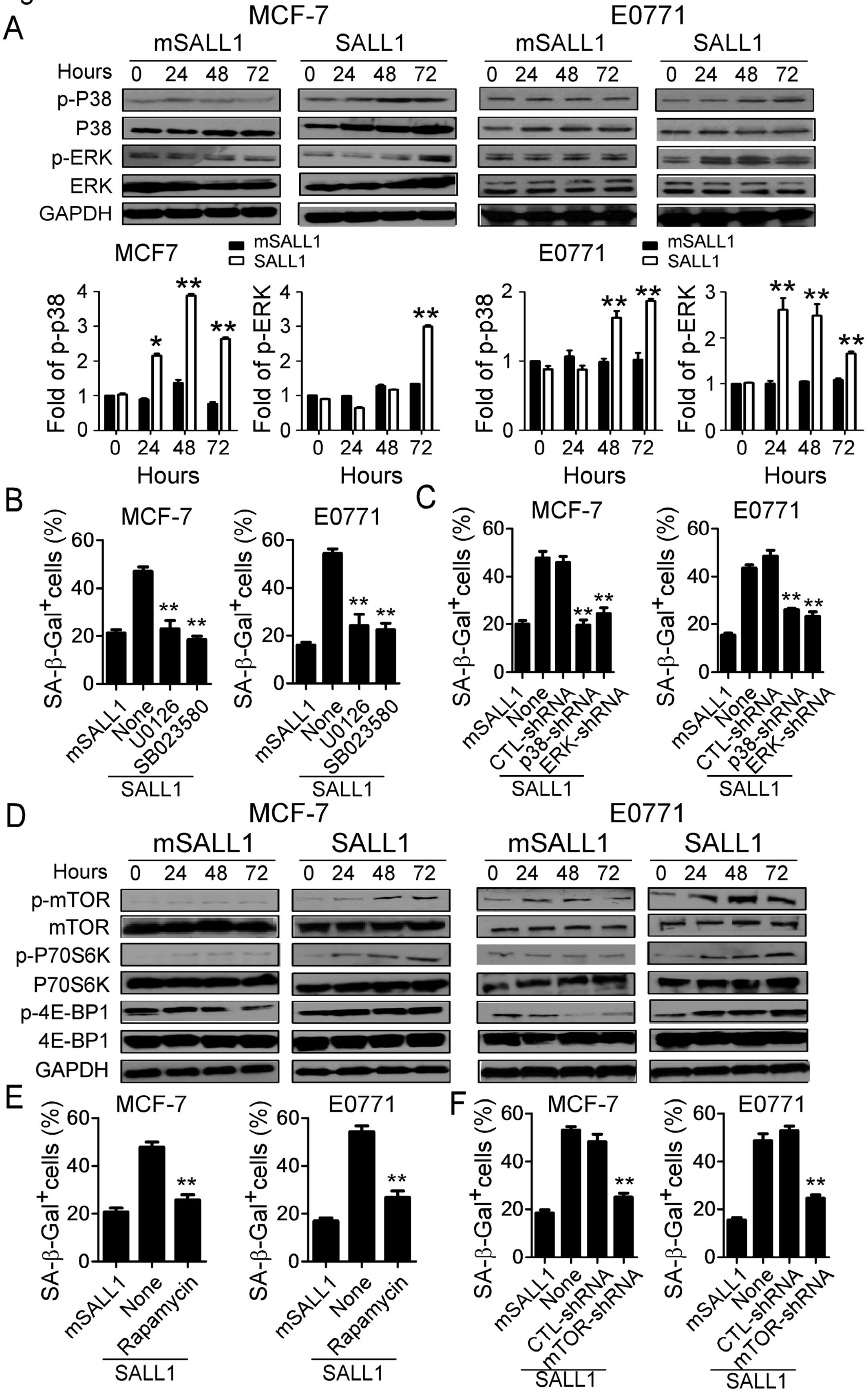

Figure 7. Ma et al.

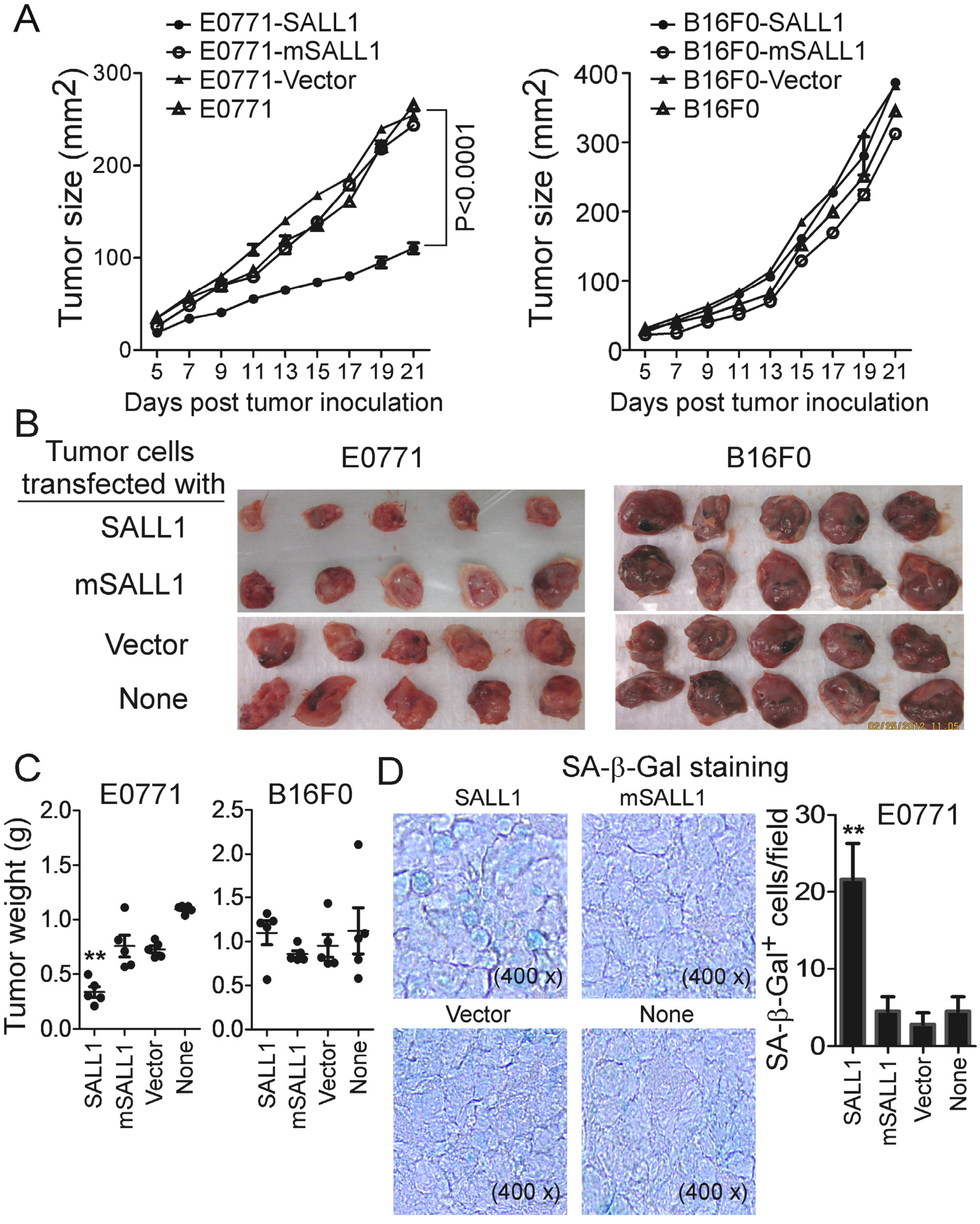

Figure 8. Ma et al.

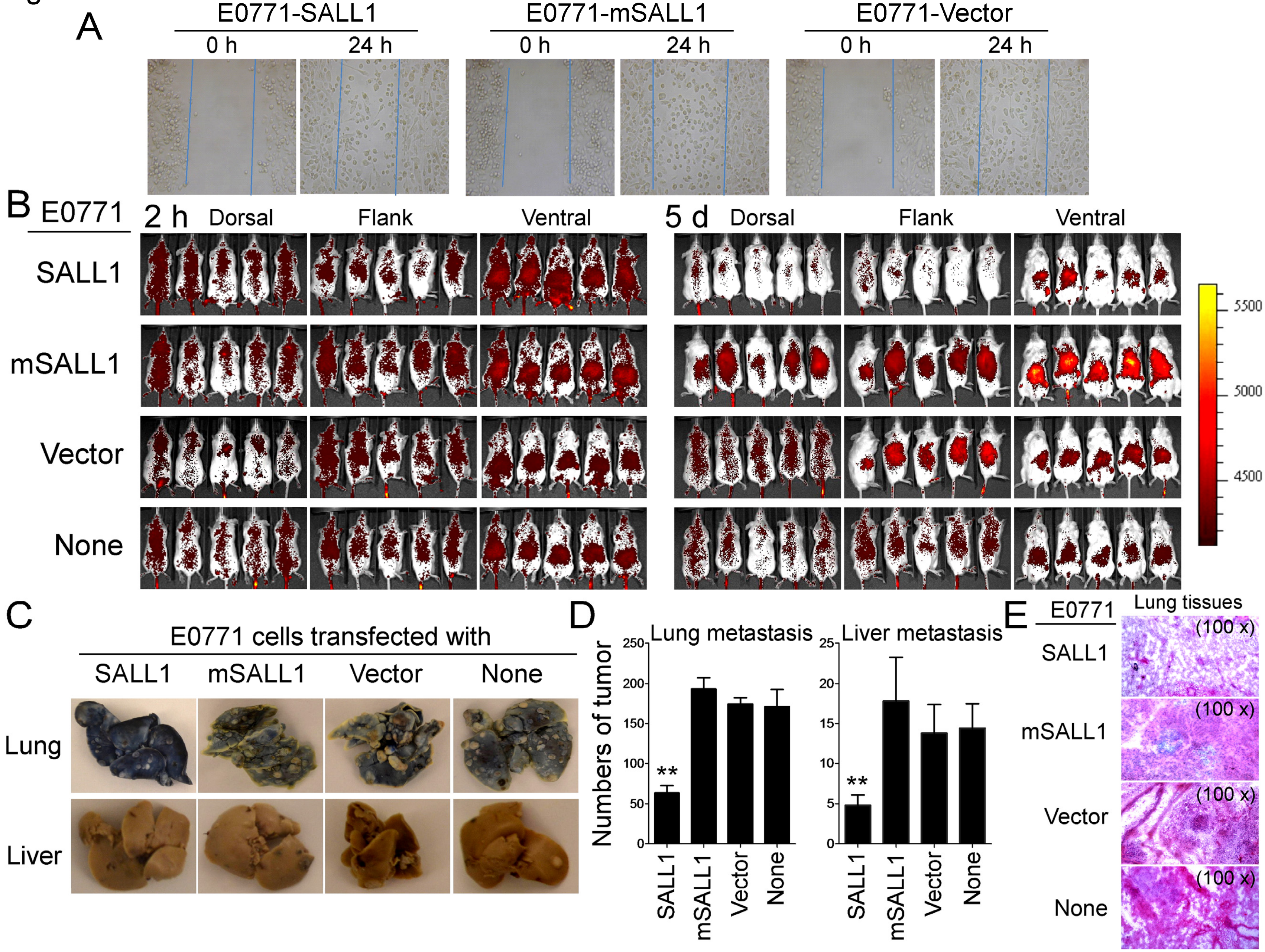

Figure S1. Ma et al.

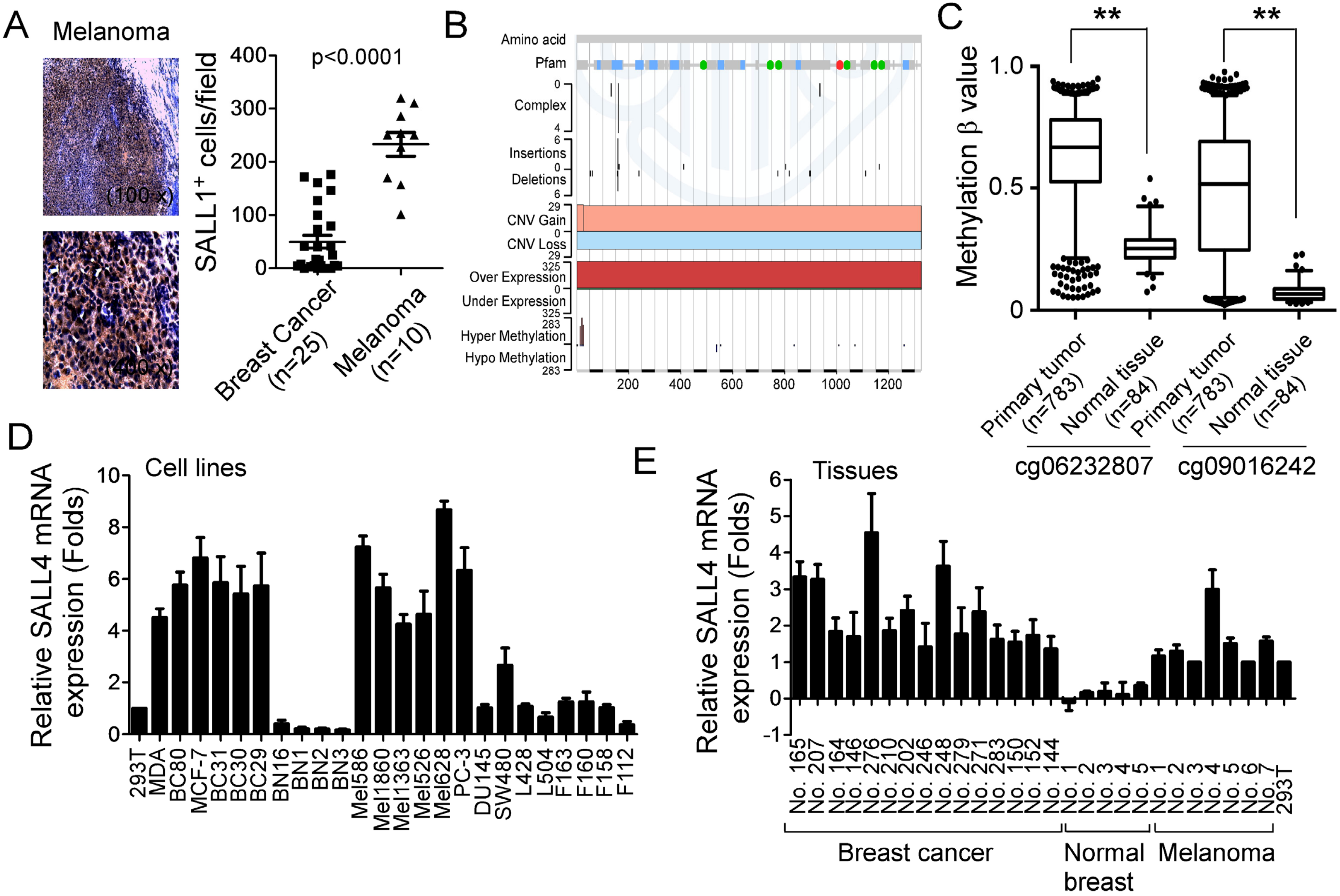

Figure S2. Ma et al.

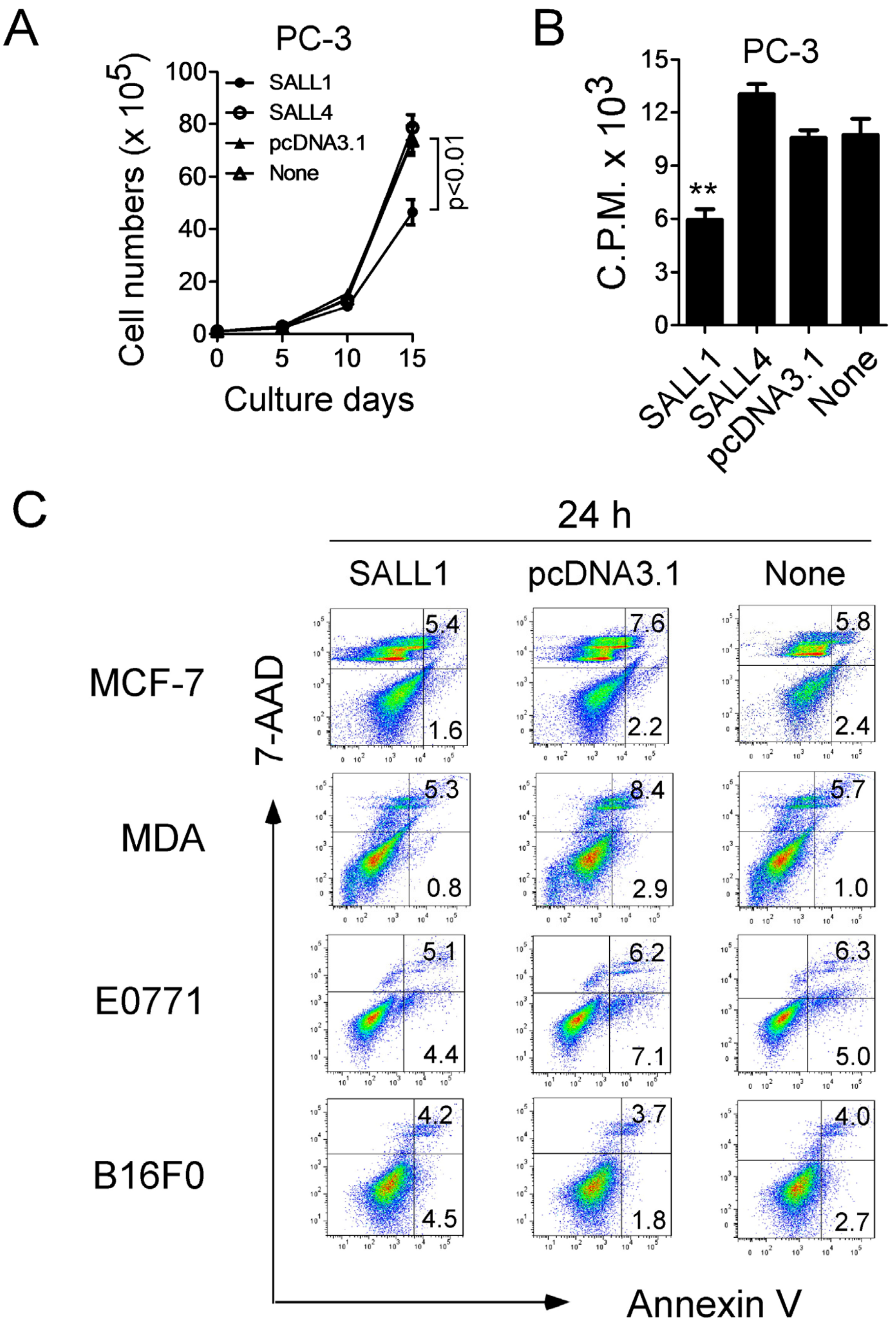

Figure S3. Ma et al.

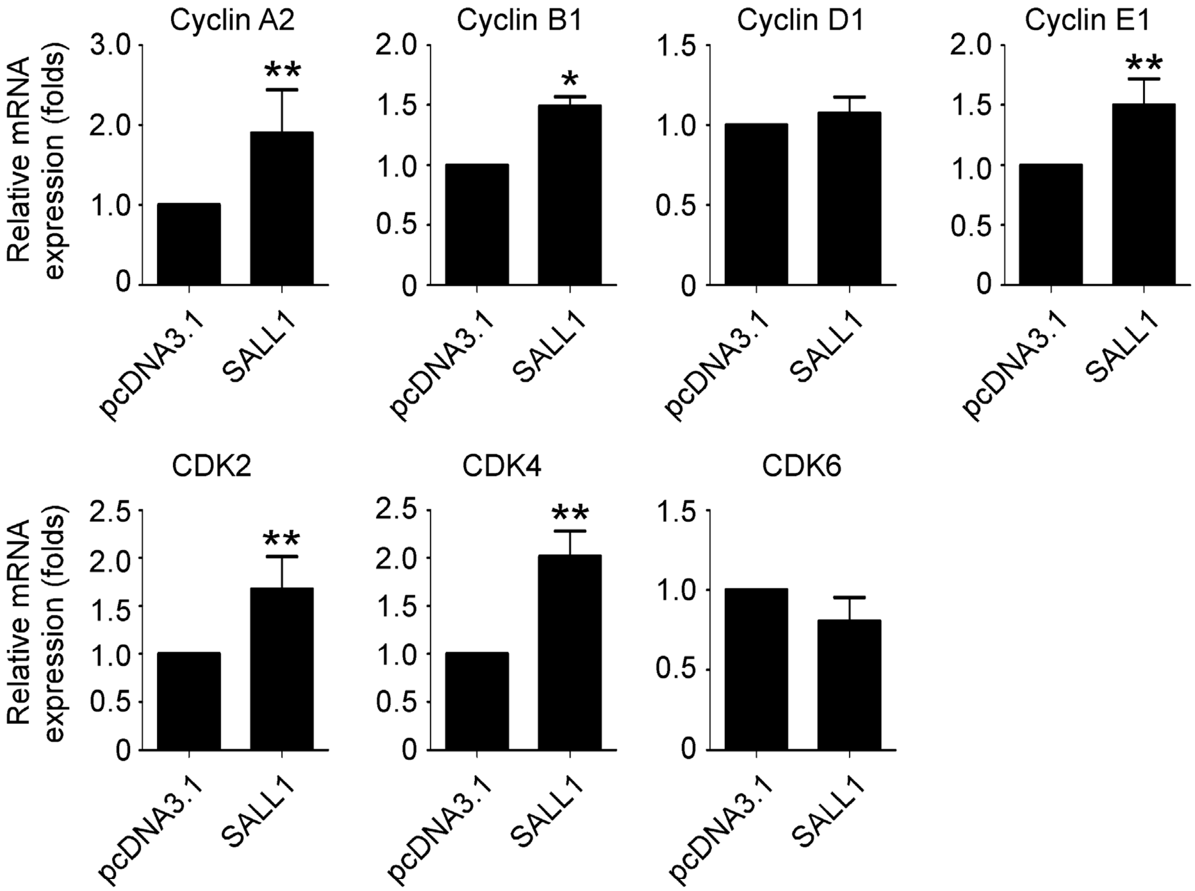

Figure S4. Ma et al.

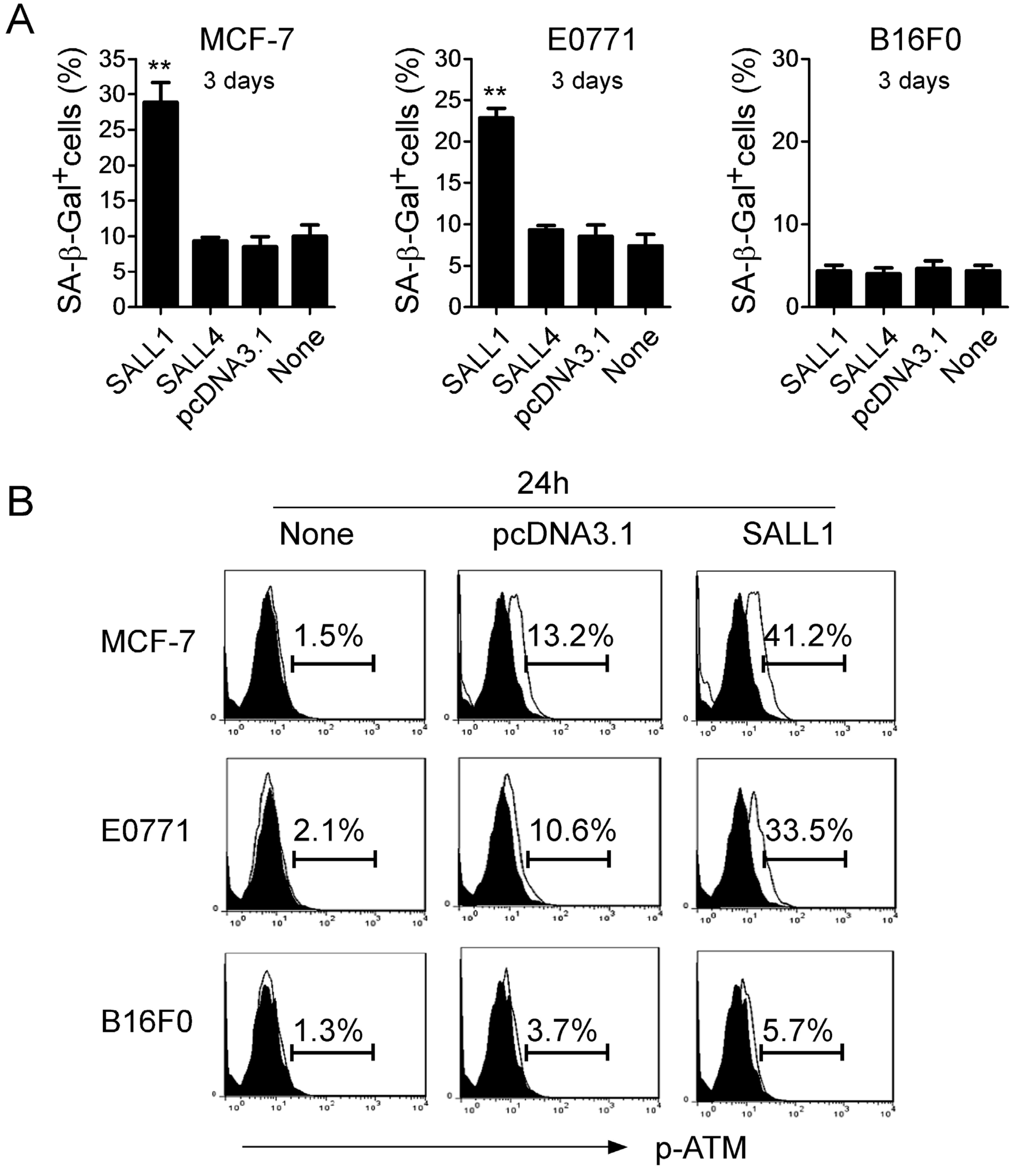

Figure S5. Ma et al.

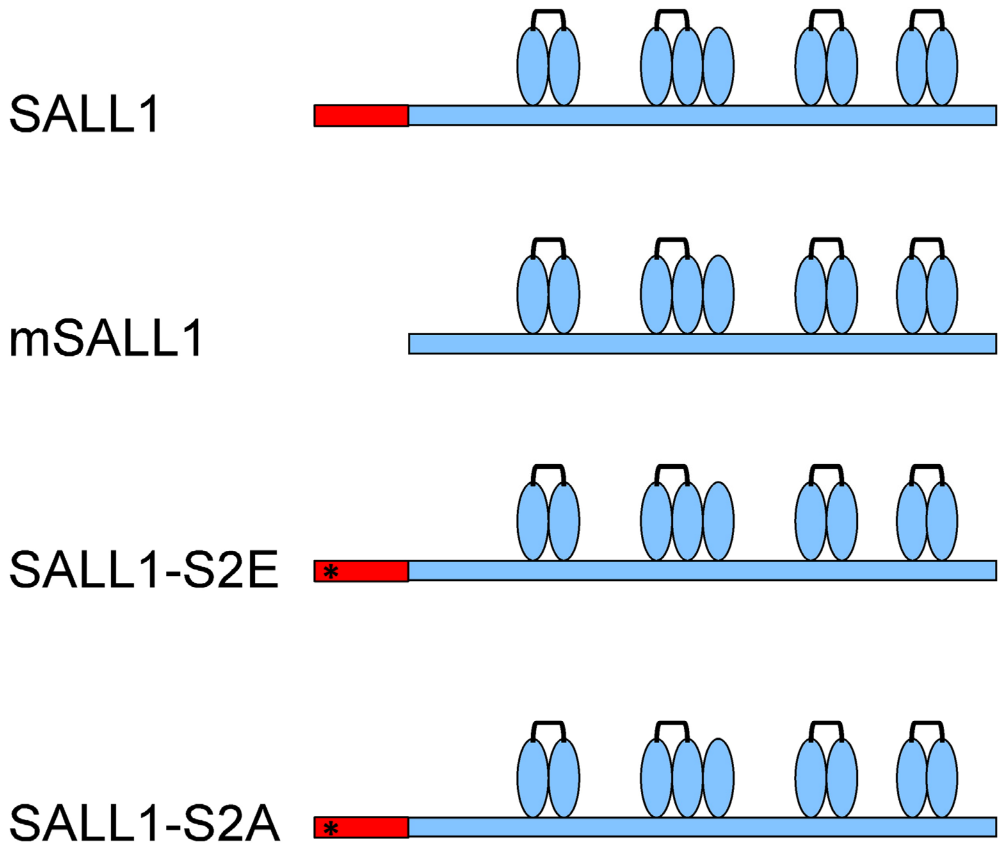

Figure S6. Ma et al.

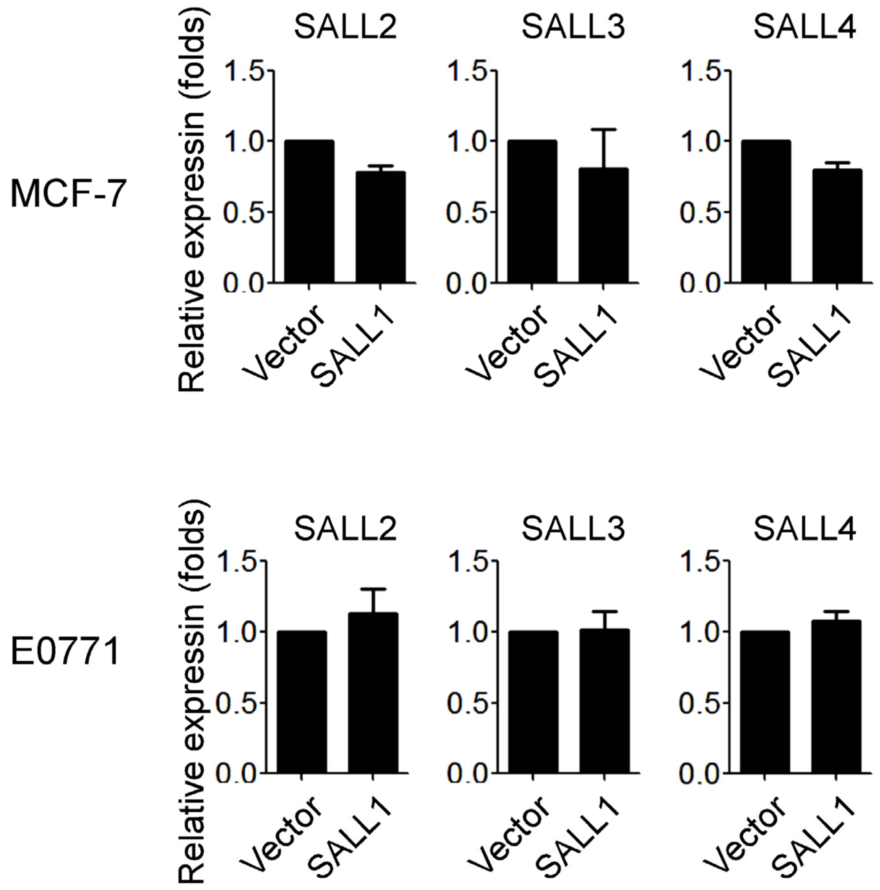

Figure S7. Ma et al.

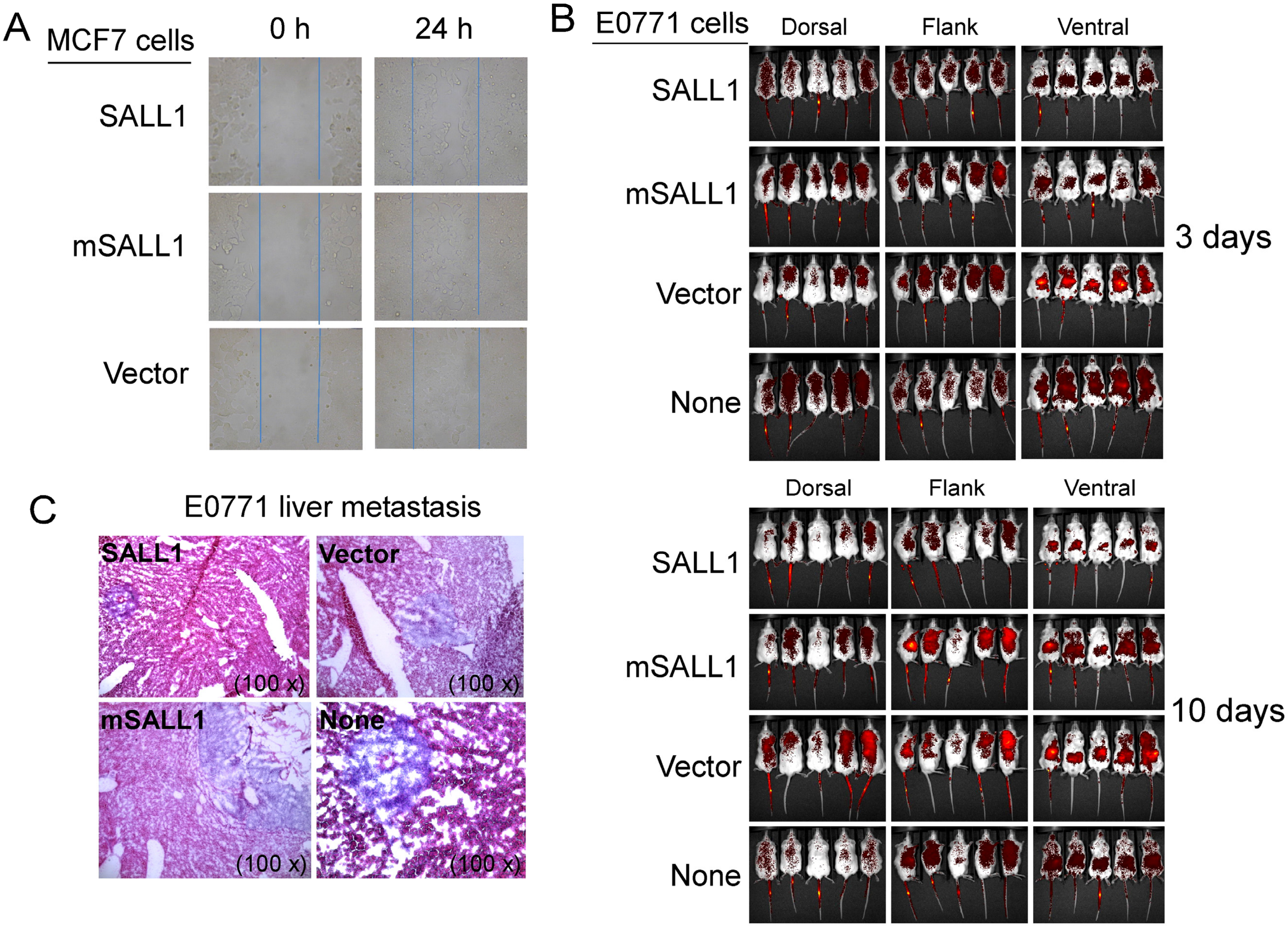

Supplement: Supplementary file 1 — Figure S1. SALL1 and SALL4 expression levels in different types of human cancers. (A) SALL1 expression in tumor cells in breast cancer and melanoma tissues was determined using the immunohistochemical staining. Numbers of SALL1+ tumor cells in melanoma tissues were much higher than those in breast cancer tissues. Expression level of each dot shown in the right panel is the average numbers of SALL1+ cells per high field (400 x) in each tissue sample. The median number of SALL1+ cells in each group is shown as a horizontal line. Significance was determined by unpaired T test. (B) The COSMIC analysis shows the mutations and the promoter methylation status of SALL1 gene in breast cancer tissues. (C) SALL1 promoter genes are highly methylated in the primary breast cancer tissues comparison with solid normal tissues. The 2 specific molecular probes which span the proximal promoter region of SALL1 gene were selected based on the information in COSMIC. The box plots showed that the methylation β value of particular promoter regions in the tissues. N indicated the number of sample size. **P < 0.01 between 2 groups with the Mann-Whitney U test. (D) and (E) Gene expression levels of SALL4 in different cancer cell lines (in D) and in tumor tissues (in E) using Real-time PCR analyses. Tumor cell lines include breast cancer (human MDA, MCF7, BC80, 31, 30, 29, 16, and murine 4 T1 and E0771), melanoma (human Mel1938, Mel1586, Mel1860, Mel1363, Mel1526 and Mel1628, and murine B16F0), prostate cancer (PC3 and DU145), colon cancer (SW480), and lymphoma (L428 and L504). Normal breast cell lines (BN6 and BN16), fibroblasts (F163, F160, F158 and F112), 293 T cells, and normal breast tissues were included as controls. mRNA levels in each cancer cell line and tumor tissue were normalized to the relative quantity of GAPDH expression and then adjusted to the express levels in 293 T cells (set as 1). Results shown in the histogram are mean ± SD from three independent experiments. Figure S2. T [file 12943_2018_824_MOESM1_ESM.pdf]
